# Prices for veterinary care of dogs, cats, and horses in Sweden and Norway: comparisons between corporate chain, government-run, and independent clinics

**DOI:** 10.3389/fvets.2025.1544996

**Published:** 2025-04-17

**Authors:** Agneta Egenvall, Paul S. Valle, Ruben Hoffman, Odd V. Höglund, Anna Byström, Cecilia Lönnell, Brenda N. Bonnett

**Affiliations:** ^1^Department of Clinical Sciences, Faculty of Veterinary Medicine and Animal Husbandry, Swedish University of Agricultural Sciences, Uppsala, Sweden; ^2^Spillfree Analysis AS, Trondheim, Norway; ^3^Department of Economics, Faculty of Natural Resources and Agricultural Sciences, Swedish University of Agricultural Sciences, Uppsala, Sweden; ^4^Department of Animal Environment and Health, Faculty of Veterinary Medicine and Animal Husbandry, Swedish University of Agricultural Sciences, Uppsala, Sweden; ^5^Tequi, Stockholm, Sweden; ^6^B Bonnett Consulting, Georgian Bluffs, ON, Canada

**Keywords:** animal insurance, corporate chain, inflation, veterinarian, diagnosis, procedure

## Abstract

**Introduction:**

In a 10–15-year period, veterinary clinics in Sweden and Norway, as elsewhere, have undergone widespread corporatisation. High veterinary care costs have received attention in the lay press and from competition authorities. Whether corporate chains and independent clinics differ in price levels and how clinic characteristics, such as on-call service, affect pricing is not well-documented. The aim was to analyse prices levels and price changes for various diagnoses/procedures for dogs, cats, and horses from clinics in Norway and Sweden and to examine the influence of affiliation (corporate chain, government-run, or independent), extraction date, and clinic characteristics (e.g., on-call service) on prices.

**Materials and methods:**

Data from a price comparison site were extracted five times between 2 January 2023 and 2 January 2024. Prices for procedures such as vaccinations, gonadectomy, euthanasia, emergency care, diagnostic imaging, certification, and planned surgery were included. Descriptive statistics and mixed models were used to analyse effects of affiliation (Anicura, The Swedish District Vet Officers (DV), Dyrenes venn, Empet, Evidensia, Vettris, and independent), clinic characteristics (animal hospital or not, on-call service, and number of hours open Mon-Fri), and extraction date.

**Results:**

Prices were analysed for 37 procedures (16 dogs, 11cats, and 10 horses) from 771 clinics, of which 502 (65%) were independent. Most clinics with corporate affiliation belonged to Evidensia and Anicura. In statistically significant comparisons, their prices were generally higher than those from the independent group. For Anicura, the median annual price increase (in Euro) was 8%, DV 5%, Dyrenes venn 53%, Empet 12%, Evidensia 15%, Vettris 7%, and the independent group 6%. Multivariable results generally corroborated the descriptive figures.

**Discussion:**

Targeting a range of procedures in two nearby countries, veterinary care prices varied with country, clinic characteristics, and affiliation. Clinics belonging to corporate chains charged higher prices than independent clinics. Most prices increased over the year. Possible reasons for the differences between clinics are investments in equipment or number of staff, expenditure on continued education of staff, or different demands for profit. Increased price transparency within veterinary care might reduce the impact of high prices and perhaps also limit price increases.

## Introduction

Investment companies have, in the last 15 years, entered the European veterinary sector and consolidated the market by establishing dominant chains of veterinary care providers, often referred to as “corporates.” In Sweden (SE), this trend started in 2011, and already in the following year, it was stated that many veterinary care providers were part of two major chains, Evidensia and Anicura (1). According to later investigations, these chains had a market share of approximately 40% (measured in turnover) (2). Between 2013 and 2016, the turnover of the veterinary market increased by 38%, and the Swedish competition authority concluded that price increases were ‘indeed rampant’ (2). During the same period (3–5), the public as well as insurance companies have, through media, objected to high prices as well as large price variations. Despite considerable anecdotal information and public concern about increased veterinary prices worldwide, there has been little scientific analysis of the situation.

In SE, the first animal hospital was established in 1933 (6). The perception by stakeholders was that the veterinary care sector developed rather gradually during the 20^th^ century, and in SE, there is a longstanding tradition of having pets and horses insured (7). In 2023, 95% of the dogs and 69% of the cats had health insurance (8), which is a considerably larger proportion than found in most other countries. In Norway (NO), the first private run animal hospital was established in 1953 (9). The perception by stakeholders is that the Norwegian veterinary market has developed quite rapidly during the last three decades. In NO, insurance is less widespread, with 27% of all pets insured in 2023 (10).

There has been little scientific analysis of what owners actually pay for veterinary care. A few questionnaire-based studies on pet owners’ yearly expenditure on veterinary care have been conducted in the US (11), Canada (12), and New Zealand (13). Based on insurance claims, veterinary care prices for horses in SE were found to have increased four times more than the consumer price index (CPI) (41% vs. 10%) between 1996 and 2004 (14). Lifetime care costs for dogs visiting seven veterinary clinics in Denmark were recently analysed (15). Egenvall et al. (16) described the levels and variation of prices for veterinary care for 15 months (years 2022–2024), looking at five European countries (SE, Denmark, NO, the UK, and Ireland). Veterinary care prices were extracted from the web, including from a price comparison site, VP (vetpris.se) (17). The VP site presents standardised veterinary care prices (in Swedish and SEK) for dogs, cats, and horses in SE, NO, and DK. From an interactive online map, VP provides information on which procedures each clinic performs, opening hours, and, if available, price information. Prices for elective procedures are sourced from clinic websites and stated to be updated at least monthly. Prices for other diagnoses and procedures related to the treatment or investigation of acutely or chronically ill patients are mainly based on receipts submitted by pet owners. The authors evaluated the benefits and limitations of the VP data (16). It was concluded that the VP data for elective procedures largely reflected those recorded directly from the websites of veterinary care providers. Receipt-based price information was often available for only a few clinics and procedures, and data were updated only when new receipts were submitted by owners, making it uncertain if the prices were representative at a specific point in time. Egenvall et al. (16) also collected data directly from clinic websites and identified several challenges that need to be overcome to obtain and analyse price data directly from the web in a time-efficient manner. Notwithstanding, the VP data were found to be adequate for further analysis.

The prices charged for veterinary care may be influenced by many factors ranging from clinic-specific to economic conditions in society. Reasons for veterinary care providers (from hereon called ‘clinics’ irrespective of size) to charge different prices for essentially the same procedures include differences in the level of equipment, the general competency and experience of the staff, and the service in terms of, e.g., opening hours they provide. A clinic with extensive resources may not use all these resources for a patient that responds favourably to treatment. However, if complications occur, they can offer more advanced care compared to less equipped clinics. For example, if an animal has a post-operative complication, the animal can, e.g., be monitored and treated in an intensive care unit. Clinics with extensive resources need to consider the return on investments made. Clinics also differ in accessibility, with some being open only during regular hours while others have after-hours service, which may stretch from a few hours during weekdays to around the clock all days of the week, the latter normally expected for animal hospitals. Whether clinics have in-patient care is often associated with the number of hours open. However, this is not always true; providers with ambulatory practice, such as the government-run veterinary organisation, the Swedish District Veterinary Officers (DV) (18), may be available at all hours but do not have in-clinic care during the nights. Thus, the characteristics of each clinic/affiliation are important to consider when examining prices for veterinary care.

In our previous study (16), pricing of veterinary care was analysed, but the type of clinic and whether belonging to a larger “organisation”, e.g., affiliated to a corporate chain, was not considered. Furthermore, only prices for a few procedures were analysed. The study revealed rather large between-country differences in prices for gonadectomy (GDY). This provoked the question of whether some countries have low prices for one type of procedure but high prices for another type of procedure and vice versa. Hence, it was considered paramount to study a wider range of procedures to get a more comprehensive picture. This study uses some of the same data, complemented with information based on manual audits of clinic websites, focusing on veterinary care prices for dogs, cats, and horses in SE and NO. The main aim was to study a wide range of diagnoses and procedures and evaluate how prices have changed over time and to what extent affiliation (in particular, corporate chains vs. independent clinics), extraction date, and other characteristics of the clinics (e.g., after-hours service) affected prices.

## Materials and methods

This study utilises previously published data (16), complemented by data on affiliations and clinic characteristics collected from clinic websites. As it was possible that secondary personal identifiers could be found in the raw data (e.g., if a URL was made up of a veterinarian’s name), the data were handled according to routines to preserve confidentiality at The Swedish University of Agricultural Sciences, with the protocol dictating that only two of the authors had access to the raw data. However, an ethical permit was not considered necessary for this type of study according to Swedish law.

The data were assembled through web-searching as previously described (16). The web search was initiated in autumn 2022 and was repeated five more times with approximate 3-month intervals. The current study uses data from 2 January 2023 (in this study denoted I), 13 April 2023 (II), 29 June 2023 (III), 30 September 2023 (IV), and 2 January 2024 (V). Only price data from VP (17) were used in the current study.

Data were managed and analysed in SAS (19) and MATLAB (20). Fields included extraction date, clinic identifiers (name, URLs, and numbering identifiers), procedural and diagnostic information in text and number (from VP) formats, and price information. Clinic information was downloaded from VP in July 2023, including information on hours open during each weekday. Prices in national currencies and prices converted into euro (€), using conversion rates at times of extractions (21), were used in the analyses. We cross-referenced the clinics listed at VP to those found at one of the largest company search sites (22). We found that veterinary care providers missing from VP were mainly single-vet part-time practices.

### Procedures and diagnoses

At VP, price data can be found for approximately 300 procedures or diagnoses, from hereon called procedures, and each procedure is species-specific, i.e., either for dogs, cats, or horses (17). The procedures found at VP were tabulated by category and extraction date [(16), Supplementary material]. From this list, procedures for dogs, cats, and horses related to one of the following were selected.

- Prophylactic procedures such as vaccinations and elective surgeries, gonadectomies (GDYs).
- Euthanasia.
- Acute unplanned care.
- Diagnostic imaging.
- Certifications.
- Advanced planned surgery.

In total, 37 procedures were included in the analysis. Within species, procedures that had more observations related to the above categories were selected. VP has information on what is included in a price for certain procedures. This information about each procedure was translated into English and is presented in Supplementary Table 1 (sheet ST1), together with the URLs for this information.

### Clinic categorisation

Affiliations were retrieved from the URLs using syntax for regular expressions. For the governmental ‘district’ veterinary organisation in Sweden, the word ‘distrikt’ was searched^1^ (18), ‘evidensia’ for Evidensia^2^ (23), ‘anicura’ for Anicura^3^ (24), ‘vettris’ for Vettris^4^ (25), ‘empet’ for Empet vet^5^ (26), ‘dyrenes-venn’ for Dyrenes venn (recently acquired by Evidensia, archived website https://web.archive.org/web/20221210010756/https://dyrenesvenn.no/klinikker/) (27). All other clinics were considered to be independent.

Information on hours open during each weekday was further processed using Python (28). All data were screened for reasonable durations. For each clinic, the number of hours open during weekdays was summed. This procedure yielded zero or a missing value for a number of clinics. In those cases, information on regular hours open was manually extracted from clinic websites. This frequently concerned animal hospitals that advertised themselves as open during all hours, i.e., on-call for emergency cases, but not necessarily with staff present at all times (on-call time outside regular hours was not counted for this variable). Thus, animal hospitals that advertised themselves as always on-call/open were coded as open when available for regular visits, using further information from their website. Data on the number of hours open per week were manually added or changed for 85 clinics. The number of hours open per working week was categorised as ≤35 h, 35 ≤ 45 h, 45 ≤ 60 h, and over 60 h.

Clinic websites were manually scrutinised to categorise each clinic further. Given the structure and information diversity of clinic web pages, only a few variables were considered reliable and suitable for statistical analysis. Clinics were categorised as animal hospitals if it was evident that they also had in-clinic care during the nights and were open every day for at least 12 h each day (including regular clinic-hours and on-call service). As it could not be reliably determined that a clinic did not provide in-clinic care during the nights, this information was only used to define animal hospitals, i.e., not used as an own variable. The extent to which a clinic was open for emergency care was categorised as: (i) full on-call for clinics open at all times—‘24 h all days’, (ii) extensive on-call for clinics taking part in 24 h on-call service all days of the week—‘part 24 h’, (iii) limited on-call for clinics open at least 12 h but less than 24 h all days of the week—‘>12 h all days’, (iv) weekend on-call for clinics providing emergency service during either or both of Saturdays and Sundays—‘<12 h Sat or Sun’, or (v) no on-call for clinics either not open for emergency cases at all or only during regular-hours, Monday–Friday—‘not on-call’. This coding of the clinics was initiated in February 2023 and updated/validated in February 2024. Clinics were further coded by region [län in Sweden (29) and fylke in Norway (30)].

### Statistics and modelling

Various descriptive statistics including tabulation of categorical variables and distributions (mean, SD, minima, 25, 50, and 75% percentiles, maxima, skewness, and kurtosis) for price data (most in € but some in national currency) were made for all variables stratified by procedure. Price data were plotted by procedure, extraction date, and affiliation. Four outliers were detected and deleted. These were a price of €664 for vaccination—distemper, hepatitis, parvo, and parainfluenza virus infection for dogs, €504 for vaccination—rhinotracheitis virus, calicivirus for cats, €548 for X-ray examination for hip dysplasia in dogs, and €593 for influenza vaccination for horses. Within species, mean clinic prices per procedure were compared using Pearson correlation. Price data for each procedure were evaluated in SAS using the mixed procedure. The dependent variable, price, was log-transformed, and residuals were scrutinised for normality during modelling. If model residuals deviated from normal distribution to a substantial extent, these were modelled without the affiliation DV (based on scrutiny of Pearson’s residuals, residual histograms, and quantile-quantile plots). Independent fixed effect variables included were affiliation, extraction date, hours open during weekdays (as categorised above), extent to which a clinic was open for emergency care (as categorised above), whether or not an animal hospital, country, and the two-way interaction affiliation*extraction date. Clinic, nested within the region, was entered as a random effect. Models were reduced backward from full models using global *p*-values <0.05. Multiple tests were corrected using Sidaks adjustment. Using Sidaks test, *p*-values below 1−(1−*α*)^1/m^, where α is the type 1 error rate and m is the number of different null hypotheses to be evaluated, are considered statistically significant. For the affiliation*extraction date interaction, the PLM procedure in SAS was used to find statistically significant comparisons (at *p* < 0.05). Pairwise comparisons were evaluated for between-affiliation within the extraction date. Time effects were mainly evaluated by comparing the first and last dates within affiliation (though other time effects have also been included in the results). For six procedures, there were data from only one clinic within an affiliation. Since the primary research question regarding affiliation was comparing corporate chains vs. independent clinics, this was handled as follows when possible: if the clinic was affiliated with a larger corporate chain, it was reallocated to another big corporate chain, and otherwise to the independent group, for the procedure in question. This could be implemented for four of the six procedures. For GDY in female and male dogs, data for “Dyrenes venn” were reallocated to independent affiliation. For ophthalmic examination with certificates in dogs and dental extraction in cats, data from Vettris were reallocated to independent affiliation. For oral examination and treatment and tetanus vaccination of horses, data from Anicura were reallocated to affiliation “Evidensia.” In the other two cases, data for those clinics were not used.

## Results

### General

Data from 771 clinics were included in the analysis. The distribution of clinic characteristics by affiliation is presented in Table 1. Anicura, Evidensia, and the independent affiliation had clinics in all categories, while the Swedish government-run DV [DV, (18)] and smaller affiliations did not. For example, 29 of the 39 (74%) animal hospitals were owned by corporate chains. Of all Anicura-clinics, 15% were animal hospitals, for Dyrenes venn 13% and Evidensia 18%. Only 2% of independent clinics were labelled as hospitals. Supplementary Figure 1 shows a map of clinic locations for the independent and corporate-affiliated clinics, respectively.

**Table 1:** The number (percentage) of clinics within affiliations by on-call category, categories for hours open Monday to Friday, animal hospital (yes/no), and country.

Variable	Category	Anicura		Districts vet		Dyrenes venn		Empet		Evidensia		Independent		Vettris		Total	
On-call category	24 h on-call all days	4	(6)	0	(0)	0	(0)	1	(8)	13	(13)	39	(8)	0	(0)	61	(8)
	On-call > = 12 h a day	6	(9)	0	(0)	1	(13)	0	(0)	6	(6)	9	(2)	0	(0)	22	(3)
	Open <12 h/day Sat and/or Sun	8	(12)	0	(0)	1	(13)	5	(42)	15	(15)	32	(6)	2	(18)	63	(8)
	Part of 24 h call all days	2	(3)	68	(100)	0	(0)	0	(0)	0	(0)	30	(6)	0	(0)	96	(12)
	Not on-call	48	(71)	0	(0)	6	(75)	6	(50)	68	(67)	392	(78)	9	(82)	529	(69)
Hours open	<35 h	3	(4)	8	(12)	1	(13)	0	(0)	11	(11)	91	(18)	0	(0)	114	(15)
	35 < =45 h	21	(31)	59	(87)	5	(63)	0	(0)	37	(36)	293	(58)	10	(91)	425	(55)
Mon-Fri	45 < =60 h	40	(59)	1	(1)	2	(25)	12	(100)	44	(43)	112	(22)	1	(9)	212	(27)
	>60 h	4	(6)	0	(0)	0	(0)	0	(0)	10	(10)	6	(1)	0	(0)	20	(3)
Animal Hospital	No	58	(85)	68	(100)	7	(88)	12	(100)	84	(82)	492	(98)	11	(100)	732	(95)
	Yes	10	(15)	0	(0)	1	(13)	0	(0)	18	(18)	10	(2)	0	(0)	39	(5)
Country	Norway	33	(49)	0	(0)	8	(100)	12	(100)	23	(23)	152	(30)	0	(0)	228	(30)
	Sweden	35	(51)	68	(100)	0	(0)	0	(0)	79	(77)	350	(70)	11	(100)	543	(70)
	Total	68	(100)	68	(100)	8	(100)	12	(100)	102	(100)	502	(100)	11	(100)	771	(100)

Based on data from a study in NO and SE, extracting data five times from January 2023 to January 2024 from a price comparison site (vetpris.se) (17, 18).

The 37 procedures studied are listed in Supplementary Table 1 (sheet ST1) together with descriptions of what is included in each and which procedures are receipt-based and web-based, respectively. Supplementary Table 1 (sheet ST2) further shows the number of clinics with price data for each procedure, broken down by country, hours open category, on-call category, whether an animal hospital or not, and affiliation. For three procedures, there were no data NO, and for all procedures, the number of observations was larger in SE than in NO.

As shown in Table 1, clinics were most frequently open Monday–Friday with 35–45 h per week (55%). Most of them were not on-call (69%). Only 5% of the clinics were animal hospitals. The distribution of clinic characteristics varied by procedure. For example, prices for TPLO were predominantly found for animal hospitals (59% of the clinics with prices for TPLO were labelled animal hospitals) [Supplementary Table 1 (sheet ST2)]. For horses, being part of 24 h on-call service was the most common category for all selected procedures. The reason for this is that the prices for many horse procedures were from DV, the government-run organisation with a main aim to cover veterinary care, including on-call service for large animals.

Across procedures, the proportion of independent clinics varied from 0 to 72%. However, it was largest in general with a median of 46%, followed by DV (0–99%, median 23%), Evidensia (0–22%, median 15%), Anicura (0–32%, median 9%), Vettris (0–9%, median 3%), and the Norwegian affiliations Dyrenes venn (0–3%, median 2%) and Empet (0–3%, median 3%) [Supplementary Table 1 (sheet ST2)]. Lack of prices for some affiliations can be due to clinics not performing a procedure, no information published on the website, or no submitted receipts. Procedures that can only be performed by government-appointed veterinarians, e.g., health certification TRACES for horses, were rarely performed by affiliations other than DV. Anicura concentrates on small animals (24) and has only two clinics for horses, whereas Evidensia has clinics for both small animals and horses. The number of observations was similar for the different extraction dates [Supplementary Table 1 (sheet ST3)].

### Prices

The distributions of clinic prices (in €) are found in Table 2. Supplementary Table 1 (sheet ST4) presents distributions in national currency and the logarithm-transformed prices in €. Mean and median prices were generally close, but as skewness and kurtosis indicated non-normal distributions, prices were log-transformed before further analysis. Supplementary Table 2 shows Pearson correlation coefficients between mean prices. These were often, but not always, rather high and significant.

**Table 2:** Price distribution, in Euro, by procedure.

S	Procedure	*N*	Mean	SD	Min	25th Pctl	Median	75th Pctl	Max	Skew	Kurt
D	Blood sample (CRP)	268	106	45	40	86	89	120	260	1.2	1.1
	Caesarean section	525	2,309	765	683	1798	2,215	2,697	5,406	0.9	2.1
	Claw injury	484	318	133	102	246	299	347	901	1.7	4.0
	Dental assess/scaling	2,128	298	90	77	235	290	354	766	0.5	0.7
	Deworming cert	1,644	26	9	5	20	24	29	88	1.8	6.3
	EU passport	2,274	60	14	21	50	60	66	176	1.4	7.1
	Euthanasia	1,375	145	51	66	123	128	153	387	1.8	3.3
	GDY bitch	1,586	685	127	398	607	673	722	1,494	1.7	6.4
	GDY male dog	1845	402	83	164	348	393	440	815	0.9	2.4
	Ophthal exam cert	361	97	30	57	76	87	107	202	1.4	1.4
	Otitis	407	161	51	55	155	159	165	444	2.0	7.2
	Pyometra	493	2017	721	666	1,535	1982	2,282	5,892	1.3	3.6
	Dental extraction	584	675	236	150	498	783	820	1,649	−0.1	1.4
	TPLO	136	4,254	837	2,710	3,678	4,152	4,907	6,307	0.3	−0.4
	Vacc distemper…	2,210	47	13	25	40	44	48	117	2.6	7.7
	X-ray hip dysplasia	1939	185	52	80	150	168	216	358	0.9	0.1
C	Abscess incl drain	378	294	110	119	220	312	324	827	2.8	10.3
	Blood sample large	210	195	75	77	135	180	247	376	0.6	−0.5
	Dental assess/scaling	1991	231	78	24	178	206	285	504	0.6	0.0
	Deworming cert	1,622	26	9	8	20	24	29	88	1.8	6.2
	Euthanasia	1,383	111	34	36	86	113	128	302	0.9	2.6
	GDY female	2,753	174	68	51	127	149	209	519	1.5	2.7
	GDY male	2,849	96	38	34	68	82	112	339	1.7	3.9
	Patella exam with cert	423	67	21	21	51	63	82	122	0.4	−0.4
	Senior check	368	181	65	69	139	162	222	541	1.2	2.2
	Dental extraction	426	494	146	131	472	494	532	1,046	0.4	3.2
	Vacc calicivirus…	1946	44	13	19	39	43	45	102	2.6	8.3
H	Euthanasia	432	355	99	169	257	356	427	874	1.3	5.8
	GDY colt/stallion	439	477	120	212	391	492	560	910	0.3	0.9
	Health cert TRACES	246	251	11	111	243	252	254	259	−9.2	119.8
	Health exam cert	517	335	123	101	234	335	500	535	0.2	−1.1
	Lameness exam initial	282	350	220	85	257	301	331	1,442	2.6	8.6
	Oral exam treat	501	223	74	76	156	229	264	380	0.2	−1.0
	Sedation	494	61	26	12	40	53	89	99	0.0	−1.5
	Vacc influenza	575	49	11	17	48	50	52	101	0.9	5.3
	Vacc tetanus	463	48	10	18	49	49	51	101	0.3	4.5
	X-ray limb 1 image	224	173	33	85	161	177	184	305	0.5	3.8

Data are from a study in NO and SE extracted five times from January 2023 to January 2024 from a price comparison site (vetpris.se). For descriptions of each procedure, see Supplementary Table 1 (sheet ST1).

Vetpris (17). NO, Norway; SE, Sweden. First column: S, species; D, dog; C, cat; H, horse; TRACES (50); GDY, Gonadectomy; TPLO, tibial plateau levelling osteotomy; skew, skewness; kurt, kurtosis-both 0 for normal distributions.

Data on prices by clinic categories and affiliations, respectively, are presented in Tables 3–5. For example, the mean price of dog euthanasia varied from €212 at Dyrenes venn to €117 at Vettris. The average difference between clinic prices at the 1st vs. at the 5th extraction date (not controlling for affiliation) varied between procedures, from a 38% decrease for sedation of horses to a 45% increase for oral examination and treatment of horses (for health certification in TRACES, the 2nd and 5th extraction dates were used to estimate the price change, as there were no data from the 1st extraction date). The median of the mean price differences was a 7% increase for dogs, 9% for cats, and 7% for horses [Supplementary Table 1 (sheet ST5)]. The median overall was an 8% increase.

**Table 3:** Mean prices (SD) converted to Euro by procedure, country, and hours open Monday to Friday.

		Country				Hours open Monday to Friday							
S	Procedure	NO		SE		< 35		35 ≤ 45		45 ≤ 60		> 60	
D	Blood sample (CRP)	122	(54)	97	(35)	75	(16)	100	(33)	110	(47)	120	(79)
	Caesarean section	1,065	(268)	2,398	(699)	1834	(837)	2,243	(639)	2,450	(792)	2,983	(732)
	Claw injury			315	(118)	242	(65)	295	(82)	388	(166)	463	(173)
	Dental assess/scaling	275	(84)	315	(88)	239	(82)	286	(79)	339	(85)	416	(59)
	Deworming cert	22	(6)	28	(10)	21	(7)	25	(8)	27	(10)	41	(12)
	EU passport	63	(12)	59	(14)	54	(12)	59	(14)	63	(13)	67	(9)
	Euthanasia	202	(61)	132	(38)	152	(53)	139	(47)	167	(61)	145	(43)
	GDY bitch	464	(137)	396	(69)	371	(88)	393	(67)	426	(95)	493	(74)
	GDY male dog	844	(242)	667	(90)	685	(120)	659	(90)	727	(172)	764	(105)
	Ophthal exam cert	142	(28)	82	(11)	118	(45)	91	(28)	103	(34)	88	(15)
	Otitis	187	(51)	163	(53)	144	(31)	163	(45)	179	(98)	182	(12)
	Pyometra	1,318	(453)	2,146	(720)	1,564	(556)	2023	(589)	2,173	(771)	2,934	(1111)
	Dental extraction	393	(235)	677	(228)	551	(257)	692	(200)	612	(244)	1,157	(641)
	TPLO			4,212	(791)	5,171	(0)	4,289	(948)	4,072	(788)	4,221	(713)
	Vacc distemper…	87	(13)	44	(07)	46	(14)	46	(11)	57	(21)	50	(11)
	X-ray hip dysplasia	233	(44)	154	(23)	187	(46)	172	(43)	206	(58)	163	(20)
C	Abscess incl drain	474	(229)	286	(90)	269	(64)	277	(73)	355	(149)	565	(289)
	Blood sample large	154	(44)	230	(78)	150	(81)	190	(65)	200	(70)	304	(53)
	Dental assess/scaling	219	(67)	240	(83)	187	(78)	216	(65)	270	(73)	365	(45)
	Deworming cert	22	(6)	28	(10)	21	(6)	25	(8)	28	(10)	43	(12)
	Euthanasia	128	(35)	109	(35)	106	(41)	112	(35)	119	(34)	122	(23)
	GDY female	244	(61)	135	(24)	168	(59)	160	(54)	204	(81)	158	(22)
	GDY male	134	(37)	76	(16)	90	(32)	88	(30)	114	(47)	89	(15)
	Patella exam with certificate	76	(21)	63	(20)	70	(28)	62	(20)	67	(19)	89	(11)
	Senior check	268	(96)	167	(50)	163	(46)	169	(52)	219	(93)	132	(19)
	Dental extraction	248	(111)	509	(131)	422	(178)	497	(106)	592	(247)	523	(256)
	Vacc calicivirus…	85	(13)	42	(6)	45	(16)	44	(11)	54	(21)	47	(7)
H	Euthanasia	299	(86)	355	(79)	348	(47)	351	(58)	391	(222)	310	(0)
	GDY colt/stallion	379	(36)	473	(105)	471	(78)	468	(96)	534	(208)	346	(0)
	Health cert TRACES			249	(17)	251	(1)	249	(18)	251	(0)		
	Health exam cert	280	(116)	332	(91)	281	(121)	345	(80)	276	(108)	259	(0)
	Lameness exam initial	169	(35)	334	(172)	292	(60)	322	(136)	476	(441)	302	(0)
	Oral exam treat	166	(87)	223	(52)	232	(73)	219	(52)	212	(72)	182	(0)
	Sedation	33	(19)	62	(22)	67	(27)	63	(22)	44	(17)		
	Vacc influenza	80	(0)	49	(10)	48	(6)	49	(11)	52	(12)	56	(0)
	Vacc tetanus	80	(0)	47	(10)	49	(5)	47	(10)	47	(17)		
	X-ray limb 1 image	208	(0)	176	(25)	170	(26)	176	(20)	190	(63)	159	(0)

Data are from a study in NO and SE extracted five times from January 2023 to January 2024 from a price comparison site (vetpris.se). For descriptions of each procedure, see Supplementary Table 1 (sheet ST1).

Vetpris (17). NO, Norway; SE, Sweden. First column; S, species; D, dog; C, cat; H, horse; TRACES (50); GDY, Gonadectomy; TPLO, tibial plateau levelling osteotomy.

**Table 4:** Mean prices (SD) converted to Euro by procedure, on-call category, and whether animal hospital or not.

		On-call category										Animal hospital			
S	Procedure	24 h all days		>12 h all days		<12 h Sat or Sun		Part 24 h		Not on-call		Yes		No	
D	Blood sample (CRP)	95	(57)	120	(77)	106	(52)	91	(19)	118	(48)	115	(69)	99	(34)
	Caesarean section	2,863	(817)	2,883	(693)	2,280	(629)	2,144	(463)	2,159	(778)	3,013	(636)	2,195	(703)
	Claw injury	373	(242)	459	(161)	314	(151)	303	(14)	307	(131)	459	(216)	304	(102)
	Dental assess/scaling	314	(106)	307	(88)	326	(90)	276	(51)	299	(94)	391	(77)	293	(86)
	Deworming cert	25	(7)	31	(13)	27	(10)	24	(3)	26	(10)	30	(10)	25	(9)
	EU passport	63	(15)	65	(15)	63	(13)	62	(7)	58	(15)	68	(14)	59	(14)
	Euthanasia	142	(47)	132	(40)	182	(73)	127	(12)	151	(56)	161	(52)	146	(52)
	GDY bitch	447	(111)	455	(112)	429	(109)	398	(41)	395	(79)	482	(94)	398	(78)
	GDY male dog	733	(187)	716	(115)	724	(175)	699	(62)	668	(126)	748	(109)	680	(125)
	Ophthal exam cert	112	(41)	93	(11)	98	(32)	136	(2)	96	(32)	103	(33)	98	(32)
	Otitis	191	(89)	146	(51)	123	(0)	160	(0)	168	(87)	224	(63)	161	(51)
	Pyometra	2,948	(1130)	2,725	(728)	1837	(278)	2081	(404)	1872	(651)	3,044	(879)	1927	(561)
	Dental extraction	648	(385)	406	(228)	737	(374)	783	(110)	495	(212)	563	(303)	669	(232)
	TPLO	4,555	(863)	4,298	(727)	3,707	(396)			3,714	(576)	4,382	(754)	3,963	(808)
	Vacc distemper…	54	(20)	49	(15)	59	(21)	46	(7)	48	(15)	56	(20)	48	(15)
	X-ray hip dysplasia	204	(53)	164	(28)	209	(51)	171	(40)	184	(52)	189	(50)	185	(51)
C	Abscess incl drain	587	(349)	361	(0)	263	(0)	263	(13)	320	(114)	646	(248)	280	(70)
	Blood sample large	194	(86)	296	(51)	214	(71)	124	(49)	194	(69)	268	(22)	190	(75)
	Dental assess/scaling	246	(85)	254	(107)	258	(73)	190	(33)	238	(82)	313	(71)	227	(75)
	Deworming cert	26	(7)	33	(14)	27	(10)	24	(3)	26	(10)	31	(10)	25	(9)
	Euthanasia	113	(22)	106	(23)	124	(46)	123	(13)	108	(41)	119	(18)	113	(36)
	GDY female	204	(84)	155	(34)	205	(79)	157	(54)	171	(64)	195	(94)	173	(65)
	GDY male	111	(42)	87	(20)	111	(42)	86	(33)	95	(37)	109	(47)	95	(37)
	Patella exam with certificate	78	(23)	85	(8)	67	(19)	103	(9)	62	(20)	78	(15)	66	(21)
	Senior check	211	(53)	145	(44)	216	(124)	158	(19)	180	(61)	203	(66)	184	(73)
	Dental extraction	836	(187)	318	(0)	371	(239)	506	(74)	446	(230)	765	(180)	485	(135)
	Vacc calicivirus…	54	(22)	46	(14)	56	(22)	46	(7)	45	(15)	55	(21)	46	(15)
H	Euthanasia	499	(271)	198	(32)	336	(29)	368	(21)	279	(70)	562	(293)	346	(55)
	GDY colt/stallion	487	(267)	505	(405)	394	(0)	502	(29)	364	(127)	644	(339)	468	(96)
	Health cert TRACES					111	(0)	251	(1)					249	(17)
	Health exam cert	318	(131)	237	(107)			385	(30)	236	(79)	397	(153)	329	(91)
	Lameness exam initial	512	(606)	195	(152)	334	(0)	311	(32)	357	(232)	687	(625)	318	(132)
	Oral exam treat	192	(80)					243	(24)	174	(70)	239	(76)	219	(56)
	Sedation	29	(10)	42	(9)	28	(0)	76	(8)	36	(17)	34	(14)	62	(22)
	Vacc influenza	45	(10)	46	(9)	52	(0)	50	(0)	48	(16)	57	(0)	49	(11)
	Vacc tetanus	41	(6)	41	(5)			50	(1)	43	(18)	46	(0)	48	(10)
	X-ray limb 1 image	212	(84)	159	(0)	110	(0)	180	(0)	161	(37)	195	(89)	176	(21)

Data are from a study in NO and SE extracted five times from January 2023 to January 2024 from a price comparison site (vetpris.se). For descriptions of each procedure, see Supplementary Table 1 (sheet ST1).

Vetpris (17). NO, Norway; SE, Sweden. First column: S, species; D, dog; C, cat; H, horse; TRACES (50); GDY, Gonadectomy; TPLO, tibial plateau levelling osteotomy.

**Table 5:** Mean (SD) converted to Euro by procedure and affiliation.

S	Procedure	Anicura		Districts vet		Dyrenes venn		Empet		Evidensia		Independent		Vettris	
D	Blood sample (CRP)	132	(53)	88	(0)					126	(71)	110	(49)		
	Caesarean section	2,870	(890)	2,266	(240)					2,598	(873)	1971	(666)	2,218	(343)
	Claw injury	496	(117)	303	(14)					391	(211)	283	(103)	320	(165)
	Dental assess/scaling	372	(71)	290	(2)	254	(61)	298	(11)	389	(77)	264	(84)	338	(84)
	Deworming cert	31	(10)	24	(0)	17	(3)	20	(2)	34	(10)	23	(8)	25	(8)
	EU passport	71	(12)	63	(0)	71	(2)	58	(3)	65	(10)	55	(15)	53	(10)
	Euthanasia	133	(13)	126	(1)	212	(19)			187	(60)	142	(55)	117	(25)
	GDY bitch	475	(82)	397	(2)	466	(0)			450	(56)	380	(89)	388	(40)
	GDY male dog	757	(106)	690	(5)	808	(0)			723	(90)	658	(153)	622	(65)
	Ophthal exam cert	99	(28)							120	(38)	93	(31)	72	(0)
	Otitis	247	(174)	160	(0)					192	(64)	155	(75)		
	Pyometra	2,293	(571)	2,189	(198)					2,631	(1119)	1794	(608)	1936	(313)
	Dental extraction	368	(275)	804	(7)					568	(280)	527	(269)		
	TPLO	4,373	(939)							4,386	(572)	3,930	(829)		
	Vacc distemper…	64	(22)	44	(0)	92	(2)			57	(19)	44	(10)	42	(4)
	X-ray hip dysplasia	216	(56)	152	(1)	216	(24)	225	(18)	191	(47)	182	(54)	163	(21)
C	Abscess incl drain	480	(195)	263	(13)					437	(255)	292	(72)		
	Blood sample large	217	(54)	126	(55)					269	(73)	171	(62)		
	Dental assess/scaling	288	(65)	194	(1)	224	(33)	268	(17)	312	(70)	206	(73)	291	(60)
	Deworming cert	30	(10)	24	(0)	17	(3)	20	(02)	34	(10)	23	(8)	25	(8)
	Euthanasia	115	(14)	126	(1)	113	(12)			129	(33)	104	(43)	79	(18)
	GDY female	243	(100)	129	(1)	226	(17)	221	(10)	177	(57)	168	(58)	132	(12)
	GDY male	133	(58)	69	(0)	121	(12)	118	(15)	101	(35)	92	(32)	76	(9)
	Patella exam with cert	85	(16)							69	(21)	64	(21)	50	(7)
	Senior check	246	(64)							183	(59)	181	(75)	151	(13)
	Dental extraction			519	(37)					672	(215)	370	(195)	968	(0)
	Vacc calicivirus…	57	(22)	44	(0)	92	(2)			55	(19)	41	(10)	39	(6)
H	Euthanasia			368	(21)					631	(199)	268	(54)		
	GDY colt/stallion			504	(23)					734	(173)	336	(75)		
	Health cert TRACES			251	(1)							111	(0)		
	Health exam cert			385	(30)					337	(119)	231	(75)		
	Lameness exam initial			313	(29)					640	(541)	323	(221)		
	Oral exam treat	155	(0)	246	(16)					273	(64)	163	(61)		
	Sedation	47	(0)	77	(6)							35	(15)		
	Vacc influenza	71	(13)	50	(0)					53	(9)	46	(15)		
	Vacc tetanus	80	(0)	50	(1)					45	(1)	41	(16)		
	X-ray limb 1 image			180	(0)					192	(73)	160	(39)		

Data are from a study in NO and SE extracted five times from January 2023 to January 2024 from a price comparison site (vetpris.se). For descriptions of each procedure, see Supplementary Table 1 (sheet ST1).

Vetpris (17). NO, Norway; SE, Sweden. First column: S, species; D, dog; C, cat; H, horse; TRACES (50); GDY, Gonadectomy; TPLO, tibial plateau levelling osteotomy.

Mean prices (€) by procedure and affiliation for the last extraction date, including the change (%) since the first extraction date, are shown in Table 6 [for prices and changes in national currencies, see Supplementary Table 1 (sheets ST6, ST7)]. The median of procedure mean prices increased for all seven affiliations between the 1st and the 5th extraction date. For Anicura, the median of the annual mean price change in € (and national currency) was 8% (11%), for DV 5% (5%), for Dyrenes venn as high as 53% (64%), for Empet 12% (20%), for Evidensia 15% (17%), for independent clinics 6% (7%), and for Vettris 7% (7%). Supplementary Figure 2 shows prices plotted over time in national currency by procedure, affiliation, and country.

**Table 6:** Mean prices (converted to Euro) at last extraction and change in percent in means between first and last extraction, by affiliation.

		Anicura		Districts vet		Dyrenes venn		Empet		Evidensia		Independent		Vettris	
S	Procedure	m	%	m	%	m	%	m	%	m	%	m	%	m	%
D	Blood sample (CRP)	128	−10	89						136	0	113	−2		
	Caesarean section	3,097	7	2,299	−12					2,948	29	2065	9	2,271	0
	Claw injury	508	−4	310						457	22	296	5	328	−1
	Dental assess/scaling	400	9	301	5	353	42	313	4	437	18	275	4	358	10
	Deworming cert	34	1	25	4	27	94	22	12	37	8	24	10	27	4
	EU passport	80	17	65	5	93	45	68	28	73	15	58	8	56	11
	Euthanasia	151	20	131	5	310	54			217	25	145	6	124	8
	GDY bitch	526	11	412	5					486	9	392	4	409	9
	GDY male dog	814	8	716	5					783	10	679	4	667	10
	Ophthal exam cert	107	14							131	25	96	10		
	Otitis	270	72	165						241	37	161	4		
	Pyometra	2,509	16	2,282	65					3,083	38	1887	8	1982	0
	Dental extraction	463	−34	833	6					664	17	539	−4		
	TPLO	4,688	5							4,550	2	4,509	12		
	Vacc distemper	68	19	46	4	97				61	27	45	5	44	6
	X-ray hip dysplasia	234	13	158	5	318	67	269	32	213	17	190	10	171	11
C	Abscess incl drain	407	−17	324						661	49	304	6		
	Blood sample large	228	2	129	−23					278	−1	170	1		
	Dental assess/scaling	316	13	201	5	265	15	281	4	358	24	216	5	322	15
	Deworming cert	33	4	25	4	27	94	22	12	37	9	24	7	27	4
	Euthanasia	128	16	131	5	177	73			142	17	108	11	84	10
	GDY female	262	10	133	5	310	40	242	12	195	13	174	10	140	8
	GDY male	140	5	71	6	177	52	131	14	111	14	96	11	79	7
	Patella exam with cert	103	27							89	32	66	11	53	19
	Senior check	269	21							210	14	186	8	157	7
	Dental extraction			508	−15					790	37	380	−4	991	0
	Vacc calicivirus…	62	26	46	4	97	NA			58	29	43	7	41	4
H	Euthanasia			370	44					646	0	273	6		
	GDY colt/stallion			512	32					765	6	345	5		
	Health cert TRACES			259	NA							111	NA		
	Health exam cert			348	49					390	18	254	15		
	Lameness exam initial			313	−29					678	6	323	−14		
	Oral exam treat	153	−7	264	69					288	10	174	12		
	Sedation	46	−6	53	−44							36	6		
	Vacc influenza	79	7	52	6					55	6	48	11		
	Vacc tetanus	79	−7	52	4					48	11	44	6		
	X-ray limb 1 image			184						196	0	163	4		

Data are from a study in NO and SE extracted five times between January 2023 and January 2024 from a price comparison site (vetpris.se). For descriptions of each procedure, see Supplementary Table 1 (sheet ST1).

Vetpris (17). NO, Norway; SE, Sweden. First column: S, species; D, dog; C, cat; H, horse; TRACES (50); GDY, Gonadectomy; TPLO, tibial plateau levelling osteotomy; NA, not applicable (no price at first extraction).

### Overall model output, country, and clinic characteristics

Model results are in Supplementary Table 1 (sheets ST8, ST9). For health certification TRACES of horses (1 of 37 selected procedures), no model could be estimated because all but one clinic performing this procedure were affiliated with DV. Hence, this procedure was omitted from further analysis. In two models (abscess including drainage for cats and vaccination—distemper, hepatitis, parvo, and parainfluenza virus infection for dogs), one or two of the clinic characteristics variables had to be removed to allow estimation of least square means for the remaining variables in the model [details shown in Supplementary Data 1].

Residual plots were considered fully adequate in 21 of 36 models (Supplementary Data 1, including code and output with residual plots for all 36 models). Of the remaining 15 models, 9 models were for horse procedures. During the year of the study (2023), DV unified the presentation of price information for all their clinics, resulting in identical prices being recorded for the same procedure for all DV clinics. DV having almost the same prices led to high kurtosis (Table 2), especially for horse procedures. The 15 models considered unsatisfactory residual distributions were therefore re-evaluated, excluding prices from DV (Supplementary Data 1 also includes the 15 models without DV), and the residual plots were then judged to be satisfactory. Accordingly, these 15 models were presented without data from affiliation DV [Supplementary Table 1 (sheets ST8, ST9)].

Country was significant in 20 models [Supplementary Table 1 (sheet ST8)]. In three models, there were no data from NO. Furthermore, for 10 procedures, data were available from only five Norwegian clinics, and country was not significant in any of these 10 models. For 12 procedures, prices were higher in NO than in SE. All these procedures were planned prophylactic procedures, e.g., male and female GDY for cats and dogs, patella examination with in cats, euthanasia of cats and dogs, EU passport for dogs, ophthalmic examination with a certificate in dogs, senior check of cats, and X-ray examination for hip dysplasia in dogs. SE prices were higher for eight procedures, specifically for deworming with a certificate for cats and dogs, dental assessment/scaling for cats and dogs, dental extraction, large blood sample of cats, and emergency procedures, i.e., pyometra and caesarean section in dogs.

Animal hospitals charged significantly higher prices for five procedures (caesarean section in dogs, otitis in dogs, pyometra in dogs and dental assessment/scaling in dogs, and abscess drainage in cats) compared to prices from clinics not labelled as animal hospitals [Supplementary Table 1 (sheet ST8)].

On-call significantly impacted prices for three procedures [Supplementary Table 1 (sheet ST8)] within a total of five comparisons. While on-call categories were not directly ordered by design, in three comparisons, on-call <12 h per day on Saturday and/or Sunday charged the highest price. For example, for euthanasia of dogs, a procedure with a large number of observations (n = 1,375), the price was higher in clinics ‘on-call <12 h per day on Saturday and/or Sunday’ [mean price from Table 4 €182/least square mean from Supplementary Table 1 (sheet ST8) €176] than in clinics with ‘part of 24 h on-call’ (€127/€123).

The number of hours open during Monday to Friday significantly impacted prices for 11 procedures. For all 35 significant pairwise comparisons, the price was lower in the categories with fewer hours open during Monday to Friday compared to clinics with more hours. For example, for deworming certificate for dogs, the price was higher in clinics open 45–60 h [mean price from Table 3 €27/least square mean from Supplementary Table 1 (sheet ST8) 24] or more than 60 h (€41/€28) during weekdays’, compared to clinics open <35 h (€21/€21).

### Models with affiliation and extraction only as the main effects

Extraction date was significant as the main effect in 13 models [Supplementary Table 1 (sheet ST8)]. In five pairwise comparisons, there was an increase in price between the first and last date of extraction (claw injury in dogs, GDY of horses, health examination with certificate of horses, and vaccination for flu and tetanus, respectively, of horses).

Affiliation was significant as a main effect in six models [large blood sample in cats, claw injury in dogs, euthanasia of horses, GDY of horses, health examination of horses, and oral examination with treatment of horses, Supplementary Table 1 (sheet ST8)]. In these models, there were nine significant pairwise between-affiliation comparisons. Of the nine comparisons, eight comparisons (from five models) were between Anicura or Evidensia and another affiliation. In all these cases, Anicura or Evidensia had the highest price. Furthermore, of the nine comparisons, seven comparisons (from six models) involved the independent group, and the independent group had the lowest price in all of these.

### Models with significant affiliation and extraction date interaction

The interaction between affiliation and extraction date was globally significant for 20 procedures. The statistically significant pairwise comparisons are found in Supplementary Table 1 (sheet ST9).

There were 83 significant comparisons between the first and last extraction date within affiliation. There was at least one significant comparison in each of the 20 models [Supplementary Table 1 (sheet ST9)]. All but one significant comparison indicated increasing prices [Supplementary Table 1 (sheet ST9)]. Altogether, the median increase in mean price was 9% based on these 83 comparisons.

There were 1,377 evaluated pairwise between-affiliation within-extraction date comparisons for 19 models [Supplementary Table 1 (sheet ST9)]. Of these, 394 were significant. Anicura and Evidensia frequently charged significantly higher prices, while the independent group seldom did. Specifically, Anicura was involved in 458 comparisons, of which 165 were significant, and Anicura charged a higher price in 161 of those 165 comparisons. Evidensia was involved in 458 comparisons, of which 151 were significant, and Evidensia charged a higher price in 138 of those. The independent group was involved in 459 comparisons, of which 185 were significant, and the independent group charged a higher price in two. DV was involved in 392 comparisons, of which 108 were significant, and DV charged a higher price in 50. Dyrenes venn was involved in 308 comparisons, of which 69 were significant, and Dyrenes venn charged a higher price in 32. Empet was involved in 240 comparisons, of which 51 were significant, and Empet charged a higher price in three. Vettris was involved in 439 comparisons, of which 59 were significant, and Vettris charged a higher price in eight. Table 7 summarises results from Supplementary Table 1 (sheet ST9) and demonstrates, by affiliation, the proportions of all between-affiliation comparisons made with a significantly higher or lower price.

**Table 7:** The number of statistically significant (*p* < 0.05) comparisons from 19 mixed models included the interaction between affiliation and extraction date predicting prices for veterinary care in Norway and Sweden in 2023.

	Affiliation B														Sum Affiliation A	
	Anicura		DV		Dyrenes venn		Empet		Evidensia		Independent		Vettris			
Affiliation A	No.	%	No.	%	No.	%	No.	%	No.	%	No.	%	No.	%	No.	%
Anicura			26	(35)	17	(31)	17	(43)	7	(7)	76	(76)	18	(20)	161	(35)
DV	0	(0)			8	(8)	7	(7)	2	(3)	28	(37)	5	(7)	50	(13)
Dyrenes venn	4	(7)	4	(8)			2	(5)	4	(7)	10	(19)	8	(15)	32	(10)
Empet	0	(0)	1	(3)	0	(0)			0	(1)	2	(2)	0	(0)	3	(1)
Evidensia	0	(0)	24	(32)	9	(17)	19	(48)			66	(79)	20	(22)	138	(30)
Independent	0	(0)	0	(0)	1	(2)	1	(3)	0	(0)			0	(0)	2	(0)
Vettris	0	(0)	3	(4)	2	(4)	2	(5)	0	(0)	1	(1)			8	(2)
Sum Affiliation B	4	(1)	58	(15)	37	(12)	48	(20)	13	(3)	183	(40)	51	(12)		

Numbers and percentages of comparisons where affiliations have higher or lower prices than other affiliations are shown by affiliation pair and overall. Above the marked diagonal is demonstrated when affiliation A has a more significant comparison than affiliation B. The denominator for the percentages contrasting two clinics is based on the total number of comparisons made between these two clinics in 19 models (total number of comparisons 1,377). Marginal denominators are based on the total number of comparisons involving a particular affiliation. Details on the model comparisons are found in Supplementary Table 1 (sheet ST1).

DV, Swedish District Veterinary Officers.

Adjusted least square mean prices are found in Supplementary Table 1 (sheet ST9). For example, for caesarean section in dogs, there were nine significant between-affiliation within-extraction date comparisons [Supplementary Table 1 (sheet ST9)]. All five within-extraction date comparisons between Anicura and the independent group were significant, and Anicura had the higher price in all cases. Similarly, three comparisons between DV and the independent group were significant, and DV had the higher price in all cases. Table 5 shows the raw mean prices (ignoring extraction date) and provides a comprehensive overview while presenting a similar picture as the model results [Supplementary Table 1 (sheet ST9)]. The mean price for caesarean section in dogs was €2,870 at Anicura, €2,266 at DV, and €1,971 for the independent group (Table 5). This equates to a 46% higher price for Anicura than for the independent group ([€2,870–€1,971]/€1,971) and 15% higher at DV than for the independent group (€2,266–€1,971/€1,971, Table 5). Another example, for X-ray examination for hip dysplasia in dogs, there were 39 significant between-affiliation within-extraction date comparisons [Supplementary Table 1 (sheet ST9)]. All five within-extraction date comparisons between Anicura and the independent group were significant, all with Anicura showing the higher price. Similarly, all five comparisons between Evidensia and the independent group were significant, and Evidensia had the higher price in all cases. The mean price at Anicura was €216, at Evidensia €191, and for the independent group €182 (Table 5). However, the mean price at Evidensia was only 5% higher than the mean price for the independent affiliation ([€191–€182]/€182, Table 5).

Considering the significant effects of affiliation in models both with and without significant interaction with extraction date, Anicura had a higher price than the independent group for 17 procedures (significant as a main effect or for at least one extraction date in models with a significant interaction), Evidensia had a significantly higher price than the independent group for 20 procedures, and DV for eight procedures. The independent group had a lower price compared to one or more of the other affiliations for 23 procedures and a higher price for only one procedure.

## Discussion

To produce a comprehensive picture of pricing for veterinary care in SE and NO for small animals and horses, we analysed prices for a wide range of procedures and treatments of medical problems from a large number of clinics (n = 771). Prices for veterinary care across various diagnoses and species have not previously been systematically analysed in relation to possible explanatory factors such as clinic characteristics and ownership (large corporation versus independent). We found that veterinary care prices varied by country, clinic characteristics, and affiliation, and prices generally increased over the 1-year study period. In our sample, a substantial part of the clinics were independent (502 of 771 in total, Table 1), and many were smaller providers (Table 1). Our sample is likely to be representative of the structure of the veterinary care providers in NO and SE but with overweight for those with prices on their websites. The large proportion of independent clinics does, however, not contradict that the corporate chains have been shown to have 40% of the market share based on annual turnover (2), based on the fact that they own most of the larger clinics, e.g., almost all animal hospitals and also charge higher prices.

### Clinic characteristics

Three variables related to the characteristics of the clinics were analysed: whether it was an animal hospital or not (open all days of the year, for at least 12 h each day, with in-clinic care during nights), the number of hours open from Monday to Friday, and extent of on-call service. For 18 procedures (of 36 analysed), there were no differences in price across different categories for any of these variables. Animal hospital or not was a significant variable for five procedures, hours open was significant for 11 procedures, and on-call was significant for three procedures. Higher prices, e.g., caesarean section and pyometra surgery in dogs, at animal hospitals may arise from the more difficult cases that would benefit from advanced procedures and additional equipment and are more likely to seek or be referred to more well-equipped and well-staffed clinics. It is perhaps more puzzling why euthanasia of dogs was more expensive in clinics without on-call services and clinics open less than 12 h per day on weekdays than clinics with part of 24 h on-call.

There are several reasons for clinic characteristics variables potentially having a real-world effect on pricing, even if this was not found in the analysis. This can occur either from unmeasured covariates or from the too-crude categorisation of included variables (i.e., residual confounding). It is worth noting that statistical power differed across procedures; for several procedures, there were few observations. Furthermore, the dataset was unbalanced with respect to clinic characteristics. For example, only a small proportion of clinics were animal hospitals and, there were often fewer observations in the animal hospital category. Some clinic characteristics are correlated with one another. As an example, animal hospitals can, by definition, only have either full on-call or at least 12 h on-call all days and are also likely to have many hours open during weekdays. Furthermore, other variables than those examined in this study (e.g., specific advanced equipment) may also influence prices. Several variables were initially considered but eventually not included in the analysis as the information was not systematically displayed on the websites of the clinics. For example, efforts were made to document whether clinics had in-clinic overnight care, but this was unsuccessful.

### Over time and between-affiliation comparisons

Of the 37 procedures investigated, significant differences between the first and the last extraction dates were found in 20 models with a significant interaction between affiliation and extraction date [Supplementary Table 1 (sheet ST9)] and in five additional models without a significant interaction [Supplementary Table 1 (sheet ST8)]. The vast majority of prices increased between the first and the last extraction. However, comparing adjacent time points (3-month periods), both increases and decreases were found. This suggested that prices were continuously adjusted both upwards and downwards throughout the year [comparisons found in Supplementary Table 1 (sheet ST8, ST9)].

When the independent group differed significantly from another affiliation, independence was often associated with a lower price. This was evident in 19 models [Supplementary Table 1 (sheet ST9)]. The corporate chains, Anicura, and Evidensia were found to be associated with higher prices in most of the cases when significantly different from another affiliation. Thus, the results suggest that the clinic characteristics tested were less important for price levels than clinic affiliation. However, clinic characteristics varied within affiliation, e.g., Evidensia had everything from small clinics open only a few hours per week to well-equipped animal hospitals. Given the number of times Anicura and Evidensia were found to charge a higher price, the fact that clinics were affiliated with a corporate chain seems a major determinant for higher price levels in SE and NO. However, there are various possible reasons for this, other than attention to profit. Corporate chains often market their services as optimal, gold-standard care (23, 24); however, representatives for independent clinics reject the claim that corporate chains generally provide better care (31). Corporate chains are also more prominent as referral clinics, motivating higher price levels, even if primary and referral clinics exist within corporate chains and independent clinics. A further possible contributing reason for high costs will be if corporate chains recruit more skilled staff by offering higher salaries. However, based on salary statistics in Sweden, salaries for veterinarians have not increased more than for other professions during the last decade. During 2007–2023, the increase in average salaries in the private veterinary sector was 36.2% for the entire period, using data from yearly salary surveys distributed to Swedish veterinarians by the union (32).

We created groups for the affiliations to the best of our ability (i.e., for the affiliations identified, we tried to figure out if affiliations with different names were actually under the same umbrella corporate chain). Affiliations were of different types and sizes (e.g., Evidensia was represented by 102 clinics and Dyrenes venn by eight clinics), and ‘affiliated’ clinics may have more or less autonomy in matters related to pricing, e.g., setting prices or transparency of pricing on websites. Across procedures and when evaluated in €, DV, independent clinics, and Vettris had the smallest median percentage increases over the year (5–7%), followed by Anicura 8%, Empet 12%, Evidensia 15%, and Dyrenes Venn 53% [Supplementary Table 1 (sheet ST6)]. During data analysis, we learned that Evidensia was in the process of acquiring Dyrenes venn during the year of study, but we were unable to find out exactly when clinics were taken over (33, 34). Albeit based on only a few clinics, it is noteworthy that the clinics linked to URLs with ‘Dyrenes venn’ raised their prices drastically (in € 53%) during the year (Table 6). This may be an effect of becoming incorporated into Evidensia. We also noticed that a new corporation entered the Swedish market during the study period, acquiring 24 clinics throughout the year (35). However, it was again not possible to determine exactly when ownership was transferred, and this affiliation was therefore ignored in the analysis. From the perspective of the animal owners, while most clinics in NO and SE with corporate affiliations advertised this clearly on their websites, it can be difficult to verify that a clinic is independently managed. Furthermore, recent changes in ownership may affect pricing before being announced to the public. Furthermore, while Swedish and Norwegian clinics owned by corporate chains, especially those owned by Anicura and Evidensia, openly advertise their affiliation, both on websites and at the clinics, this is not always the case in other countries (36).

When discussing price changes in percent, it should be kept in mind that the same percentage change implies a larger numerical increase in price for a higher price than for a lower price, and while fewer animals require expensive procedures, these price increases can have a large financial impact on the owners of those animals. It should also be remembered that larger clinics see more patients per clinic. Of the clinics included in the study, only 25% in NO and 21% in SE were affiliated with Anicura and Evidensia, but these chains have large market shares [approximately 40% of the market (2)] (Table 1). From the perspective of the animal owners, it is also important to consider that payments were made in national currency while in the analysis, price changes were measured in €, which is affected by the exchange rates. Hence, the analysis was also conducted based on prices in national currencies. However, comparing the price changes in € (Table 6) and national currencies [Supplementary Table 1 (sheets ST6, ST7)], results are similar, even if price changes in national currencies tended to increase more. Thus, the approach to using € provides slightly more conservative results than using national currencies (i.e., converting prices to € has not exaggerated the price increases).

### Country

Price data were collected from 228 clinics in NO and 553 clinics in SE, numbers reflecting that there are fewer clinics in NO than SE (16). For some procedures, there were no or few observations from NO [Supplementary Table 1 (sheet ST2)], especially procedures for horses. The country variable statistically significant for 20 procedures. For 12 procedures, prices were higher in NO than in SE, all procedures representing planned prophylactic procedures or elective surgeries, e.g., cat and dog GDY. For the remaining eight procedures, prices were higher in SE. These included both prophylactic procedures, e.g., deworming with a certificate in cats and dogs, and emergency procedures, e.g., pyometra and caesarean section in dogs. Even if price observations from NO are fewer and for procedures with low numbers in NO possibly less representative, this suggests that price development has not been identical between the countries. Two to three decades ago, NO had a less developed veterinary care service compared to SE and both previously and currently a much lower insurance coverage (10). This may suggest that prices for emergency procedures have not yet caught up with the level in SE, where a large number of emergency procedures for dogs and cats are covered by insurance. However, Norwegian clinics have high prices for prophylactic procedures and elective surgeries, e.g., cat and dog GDY, where median prices are higher in NO than SE (16) (Table 3). It should be noted that comparisons of prices in NO and in Sweden were complicated by fluctuating exchange rates and rather large differences in cost of living, with NO being more expensive. Specifically, the Norwegian currency depreciated during 2023 but regained strength by the end of 2023. Furthermore, the availability of veterinary clinics and cost of living varies within the country, particularly when comparing the larger cities and the northern regions; this was accounted for as a random effect. From a wider perspective, the veterinary care sector in SE and NO are still quite similar. It has been suggested that the European market for veterinary care can be split into two areas, one including the Scandinavian countries, the UK, and the Netherlands, where there are few actors and a market dominated by corporate chains, and another including Germany, France, and Spain with a larger proportion of independent clinics (37). As SE and NO are Scandinavian countries, much bigger between-country differences would be expected if compared to countries in the latter group or worldwide.

### Procedures

To produce externally valid findings, this study strived to include several different procedures, ranging from simple prophylactic procedures to emergency surgeries. The procedures found to have the largest price increases were oral examination and treatment of horses (45%), health examination of horses with certificate (35%), and euthanasia of horses (31.5%). The procedures with the largest decrease in prices were sedation of horses (38%), initial lameness examination of horses (21%), and blood sampling and analysis of C-reactive protein for dogs (19%). It is noteworthy that all these procedures, with the largest changes, have relatively few observations.

The prices for emergency procedures are taken from owner-submitted receipts, and we have shown that prices from receipt-based procedures from VP can be outdated at times, even though VP is recently launched (16) [Supplementary Table 1 (sheet ST1) for which procedures are receipt-based and web-based, respectively]. The emergency procedures, pyometra and caesarean section in dogs, during regular hours saw 18 and 8% median price increases, respectively (calculated in €). The price for treating a cat with an abscess decreased by 7%. Instead, examining data for the web-based procedures, we see that prices for euthanasia increased considerably for all species, from 10% for cats to 32% for horses. Euthanasia of dogs and cats is a procedure with a large number of observations, and the listed prices are frequently updated by VP (weekly or at least monthly), mainly based on information from clinic websites (16). In summary, while some outliers can be found for procedures with few observations, the data do not suggest that there were any major general differences in price changes between emergency and planned care or between web-based and receipt-based prices.

The debate on veterinary care prices has been intense in Swedish media during 2023 and 2024 (4, 6, 38). Agria insurance has shown that the mean increase in costs per claim over the last 5 years was 30–41% from Anicura and 48–56% from Evidensia (39). At the same time, Evidensia has stated that their price increases were ‘only’ 4.3–4.4% per year (38). A 4.4% yearly increase is equivalent to 24% over 5 years. In general, to evaluate figures on price increases, e.g., by affiliation or clinic, it is paramount to know how these calculations were made. Such details are seldom presented when those figures are brought forward in lay media. However, even if we included many procedures, and our calculations are considerably more transparent than reports by other actors, e.g. (38, 39), our estimates of prices and price changes for different procedures cannot be used to infer how much owners spend on veterinary care in total. A few questionnaire studies have examined how much owners spend on veterinary care (11–13), albeit such results are prone to recall bias, and available estimates based on data from veterinary clinics did not account for the fact that owners may visit multiple care providers (15).

### Levels of care

For each procedure, the same standard routine treatments were included in all clinics, whether they were single-veterinarian clinics or larger animal hospitals. However, one reason for varying prices can be that clinics provide different levels of care, often based on expertise and equipment. It is often argued by stakeholders (40) that owners, considering their animals to be family members (41), drive the delivery of veterinary medicine by demanding a level of care for their animals that is as good or better than that provided to humans, and that is more or less instantly available. On the other hand, many owners are satisfied with, or have chosen, a lower level of care for various reasons (42), e.g., based on previous (negative) experiences with certain procedures. Those owners can, however, become quite overwhelmed if persuaded to choose care considered ‘gold standard’ for an ill animal. One reason veterinarians may promote gold-standard care to owners, perhaps especially in SE, is the ease with which owners can file complaints against a veterinarian on-line when the clinical course does not result in the expected outcome. Defensive medicine, i.e., to do more investigations than strictly needed, is a well-known effect when practitioners are constantly aware of the risk of complaints (43). In SE, the number of filed complaints related to dogs and cats approximately doubled between years 2018 and 2019 [personal communication (44)]. Part of this increase may be related to the perceived high prices, even if the Norwegian and Swedish complaints committees will not evaluate pricing (44, 45). Furthermore, stakeholders even argue that more and more animals are not brought to the veterinarian in time, most likely because of high prices, thereby increasing risks to the animals and possibly requiring more advanced care when finally brought to veterinary attention (46). One solution would be for veterinarians to practice spectrum of care medicine (47) while selecting investigations and treatments, using a strategy acceptable to both the owners’ cost tolerance and the animal’s problems. In theory, this can be done with clear informed consent. However, in SE, this may require meticulously formulated verbal and written agreements with the animal owners, including what and why certain actions are decided on, and this agreement would be regarded as contractual agreements. In the long run, this may provide better and more cost-effective care for more animals.

### Transparency of pricing

The need for increased price transparency has been raised in a Swedish governmental investigation (48). Following this report, DV (18), in July 2023, started publishing their complete price list on the web. Overall, prices for prophylactic care have become quite transparent in SE and NO, as well as in Denmark. Achieving transparent pricing for unplanned and emergency procedures may be more challenging for several reasons. Still, such price transparency could possibly be implemented on a voluntary basis, in collaboration with various stakeholders (e.g., animal owners, veterinarians, corporates, insurance companies, and kennel clubs). If achieved, this would allow owners to prepare for veterinary care expenses to a greater extent. It can be speculated whether increased price transparency in itself may curb the price increases or at least facilitate owners to potentially choose less expensive alternatives. By tradition and convenience, in many instances, clinics will not disclose prices for treatments until a diagnosis is made. Given that it is difficult to make a correct and complete diagnosis before an animal is seen and examined, it is difficult to correctly estimate the price for the treatment before examination. On the other hand, for an owner with an acutely ill animal, it can be difficult to choose a clinic with a lower price rather than continuing treatment directly at the same clinic where the diagnosis was made. Even a dialogue about pricing may be difficult as animal owners can be threatened, or even become locked out of a clinic after discussing expensive bills (49). It is sad if owners are unable to discuss pricing with clinics because of fear of being locked out.

### Limitations

DV had similar prices across clinics and published a common price list on the web halfway into the study period, which is reflected in Supplementary Figure 2, where many of the prices from their 68 clinics are the same. From a computational point of view, this created issues in the mixed model analysis. Even if mixed models are generally robust, it was decided to exclude the data from DV for 15 models. This means that prices from DV are not modelled for some procedures which DV commonly performs, such as for all horse procedures. Still, descriptive data from DV are included and provide possibilities for comparisons between DV and other affiliations. For example, at the last extraction date (Table 6), prices from DV were often between the independent group and Evidensia.

The number of clinics with price information for each procedure varied from 32 for TPLO to 620 for GDY in male cats, with a median of 127 clinics. The number of clinics included in the statistical analysis was further reduced for the 15 procedures where DV had to be dropped.

While the price information presented at VP has not been found to diverge from the actual prices on the web to any large degree (16), receipt-based prices for acute care may have lower accuracy. A validation based on 24 receipts suggested that the median for the standardised prices at VP for regular-hours pyometra in dogs was 29% lower than prices paid for actual cases (16). However, the results for the receipts-based procedures are similar to those for the planned and prophylactic procedures [see also (16)]. The country was included in all models with data from both countries. However, when the number of observations in NO was low, the country was never significant, suggesting insufficient power in these cases.

## Conclusion

Given the impact on owners and pets of prices for veterinary care, evidence-based price information is crucial. While lacking other data sources on the prices of veterinary care, we have continued using data from the VP price comparison website. In this study, targeting a range of problems in two nearby countries, we show that clinics belonging to corporate chains charge higher prices than independent clinics and that over the study period, they increased their prices more, both in monetary value and in percentage, also when controlling for clinic characteristics. This was also indicated by the increase in prices when one of the small chains was acquired by a large corporate chain during the course of the data collection. Transparency of prices for veterinary care, e.g., at the VP price comparison site and on clinic websites, can make owners more aware of the prices and help them choose the level of care. Ideally, further transparency from all clinics regardless of country, including their affiliations and price changes, and sharing of data from clinics and/or insurance companies would provide more information on the problems facing the veterinary sector.

## Data Availability

The data analyzed in this study is subject to the following licenses/restrictions: this study utilizes previously published data (16), complemented by data on affiliations, and clinic characteristics collected from clinic websites. As it was possible that secondary personal identifiers could be found in the raw data (e.g., if an URL was made up from a veterinarian’s name), the data were handled according to routines to preserve confidentiality at The Swedish University of Agricultural Sciences, with the protocol dictating that only two of the authors had access to the raw data. However, an ethical permit was not considered necessary for this type of study according to Swedish law. Requests to access these datasets should be directed to agneta.egenvall@slu.se.

## Footnotes

^1^ https://distriktsveterinarerna.se/

^2^ https://evidensia.se/

^3^ https://www.anicura.se/

^4^ https://www.vettris.se/

^5^ https://www.empet.no/
