# Supplementary material for: Prices for veterinary care of dogs, cats, and horses in Sweden and Norway: comparisons between corporate chain, government-run, and independent clinics
Source: Front Vet Sci. 2025 Apr 17;12:1544996. doi: 10.3389/fvets.2025.1544996 (PMC12045027; doi:10.3389/fvets.2025.1544996)

Supplementary Figure 1. All independent veterinary clinics (A) and the veterinary clinics with affiliations (B), from a study of prices for veterinary care from winter 2022/2023 to winter 2023/2024 in Norway and Sweden.

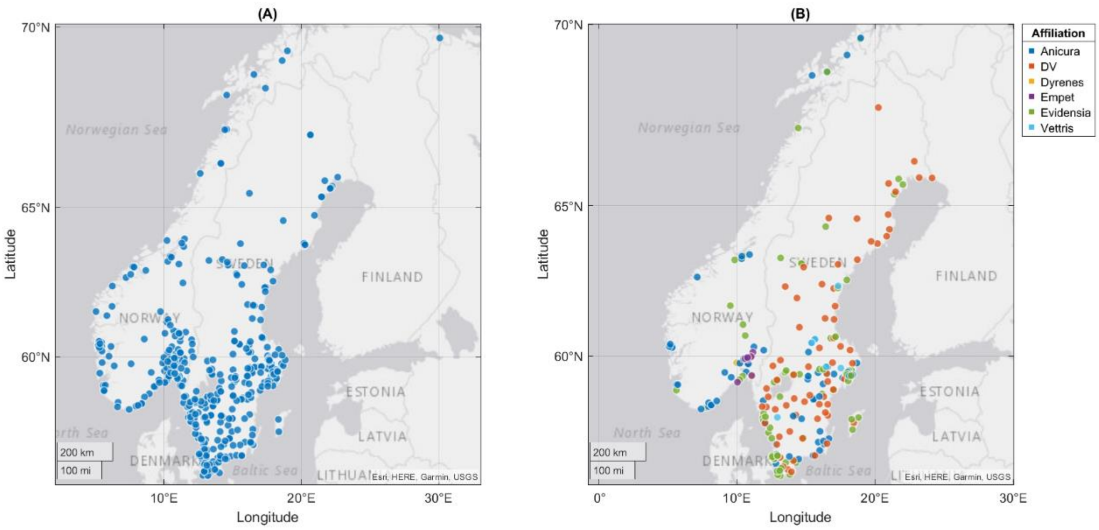

Supplement: Supplementary file 1 [file Image_1.PDF]
