# Supplementary material for: Prices for veterinary care of dogs, cats, and horses in Sweden and Norway: comparisons between corporate chain, government-run, and independent clinics
Source: Front Vet Sci. 2025 Apr 17;12:1544996. doi: 10.3389/fvets.2025.1544996 (PMC12045027; doi:10.3389/fvets.2025.1544996)
Supplement: Supplementary file 2 [file Image_2.PDF]

Supplementary Figure 2. Prices in national currency by procedure, extraction date and affiliation. Norwegian prices are found in red and Swedish in blue. Extractions were done 5 times, approximately every three months, during winter 2022/2023 - winter 2023/2024.

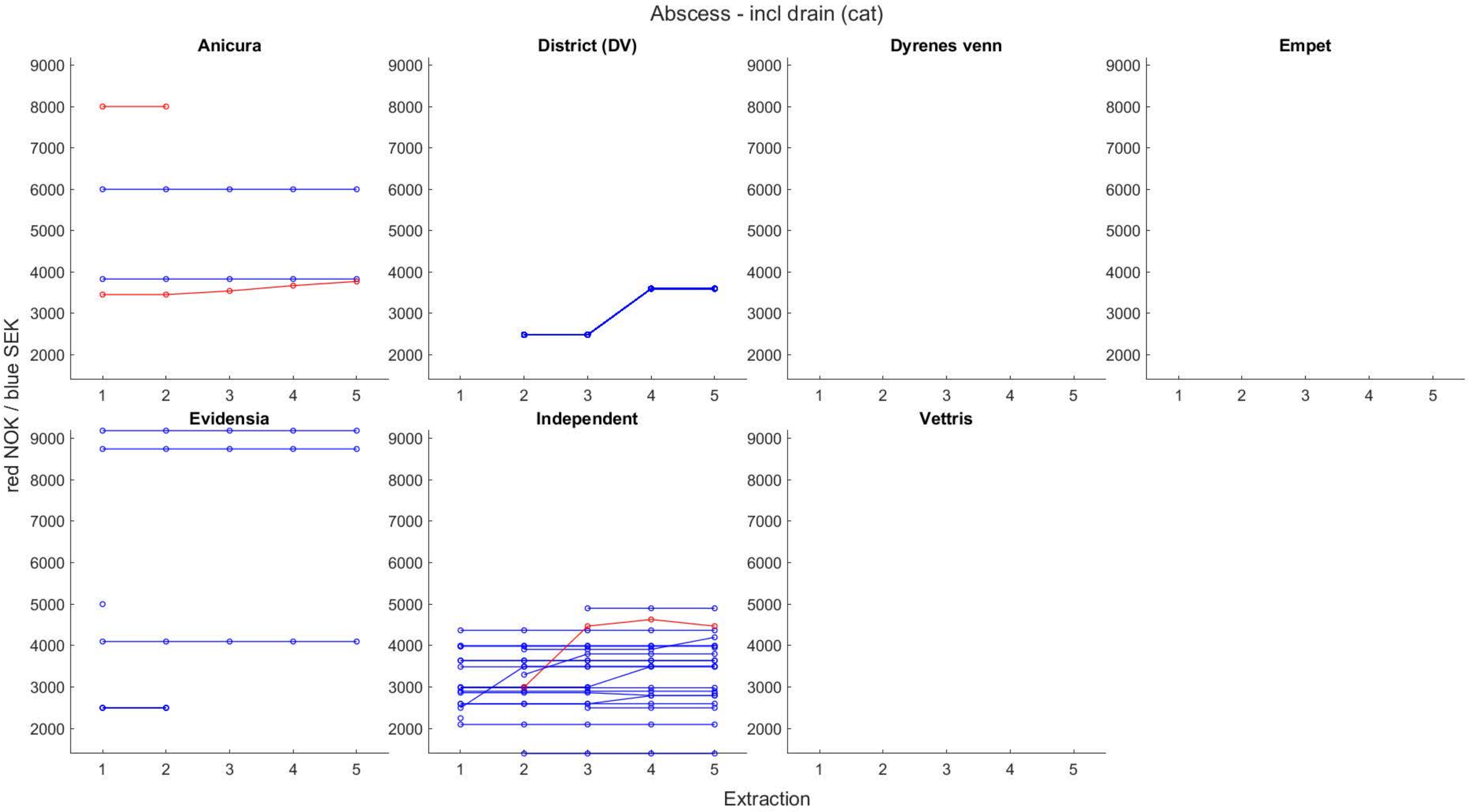

Abscess - incl drain (cat)
Anicura
District (DV)
Dyrenes venn
Empet
Evidensia
Independent
Vettris
red NOK / blue SEK
Extraction
9000
8000
7000
6000
5000
4000
3000
2000
1
2
3
4
5

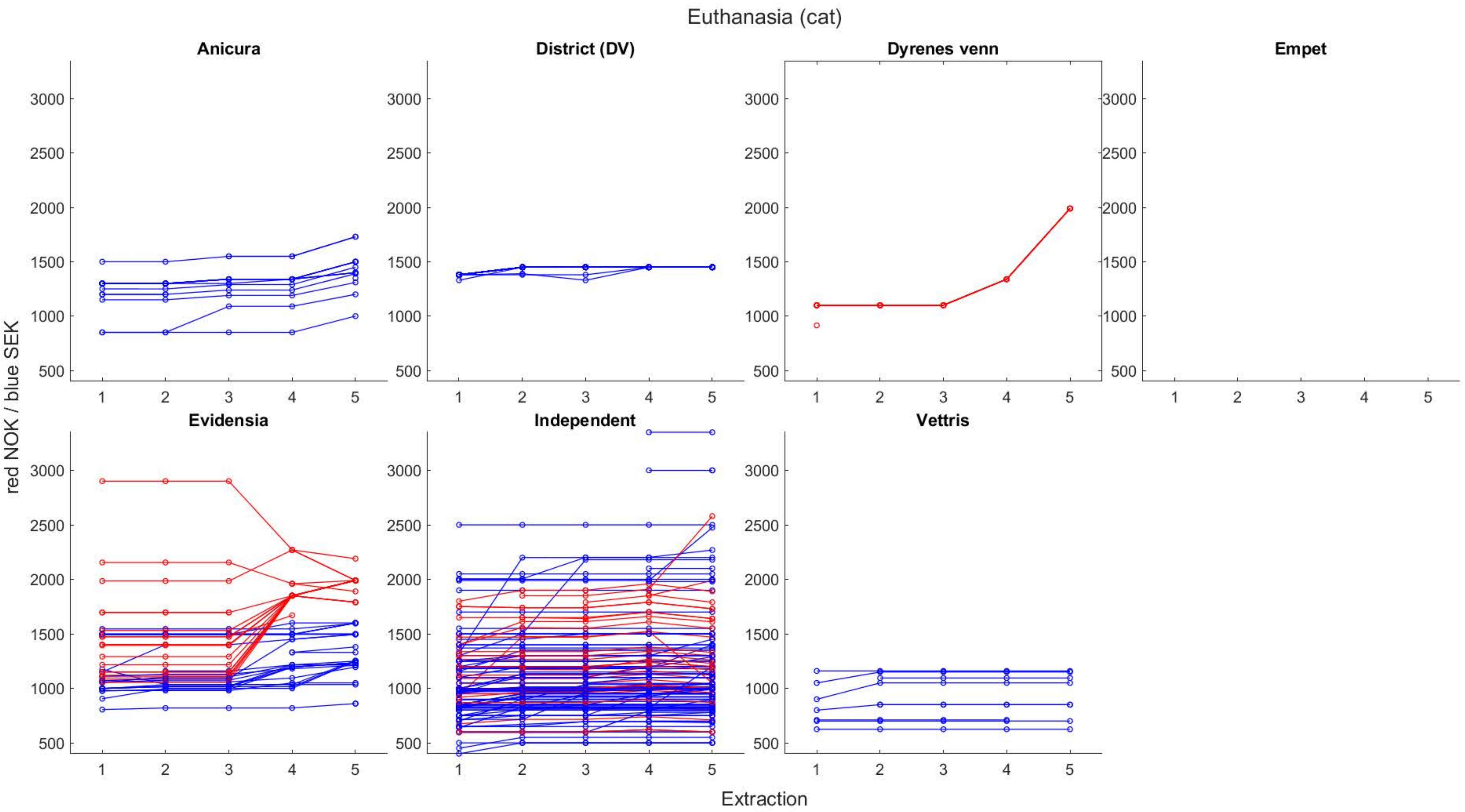

Euthanasia (cat)
Anicura
District (DV)
Dyrenes venn
Empet
Evidensia
Independent
Vettris
red NOK / blue SEK
Extraction
500
1000
1500
2000
2500
3000
1
2
3
4
5

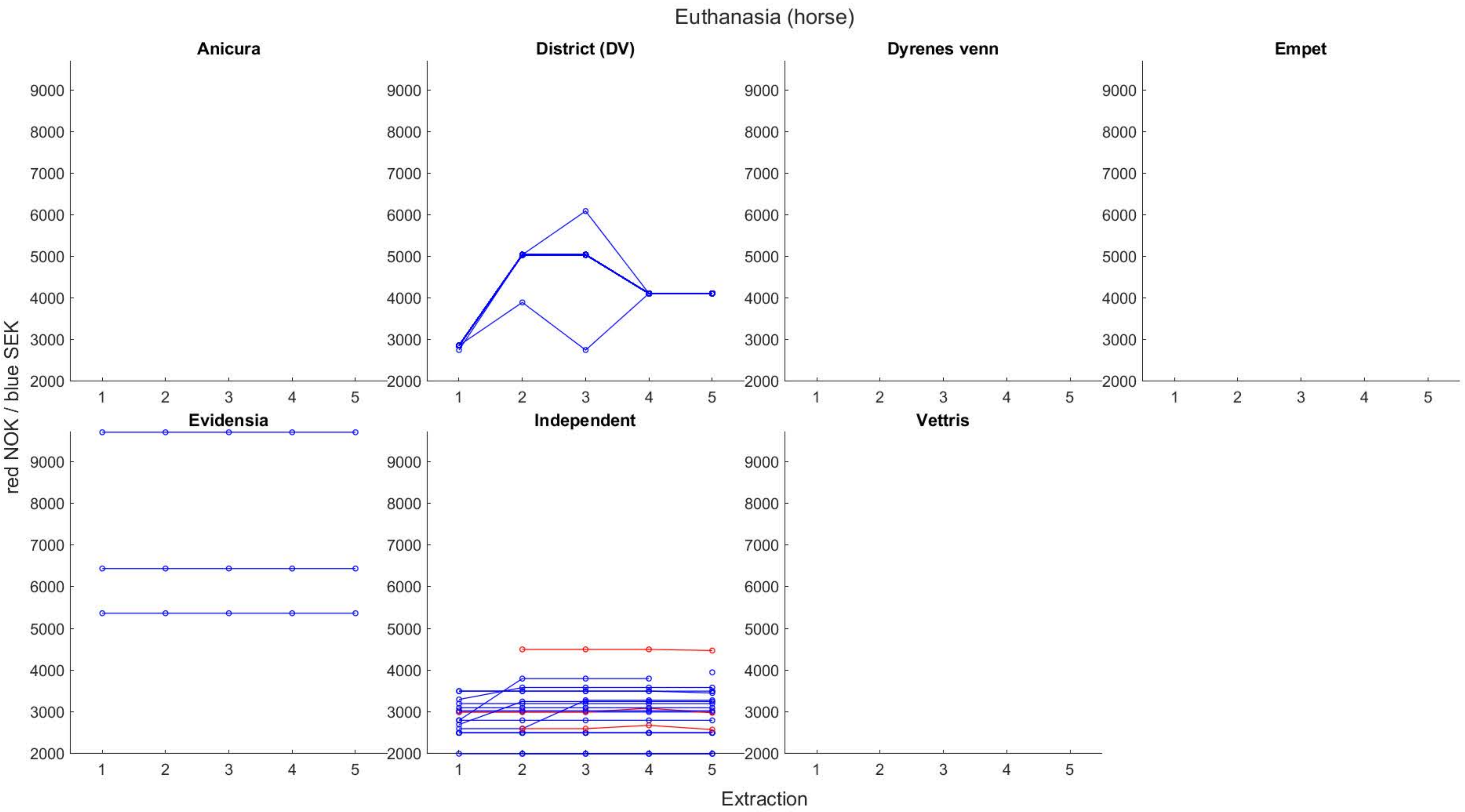
Euthanasia (horse)
Anicura
District (DV)
Dyrenes venn
Empet
Evidensia
Independent
Vettris
red NOK / blue SEK
Extraction

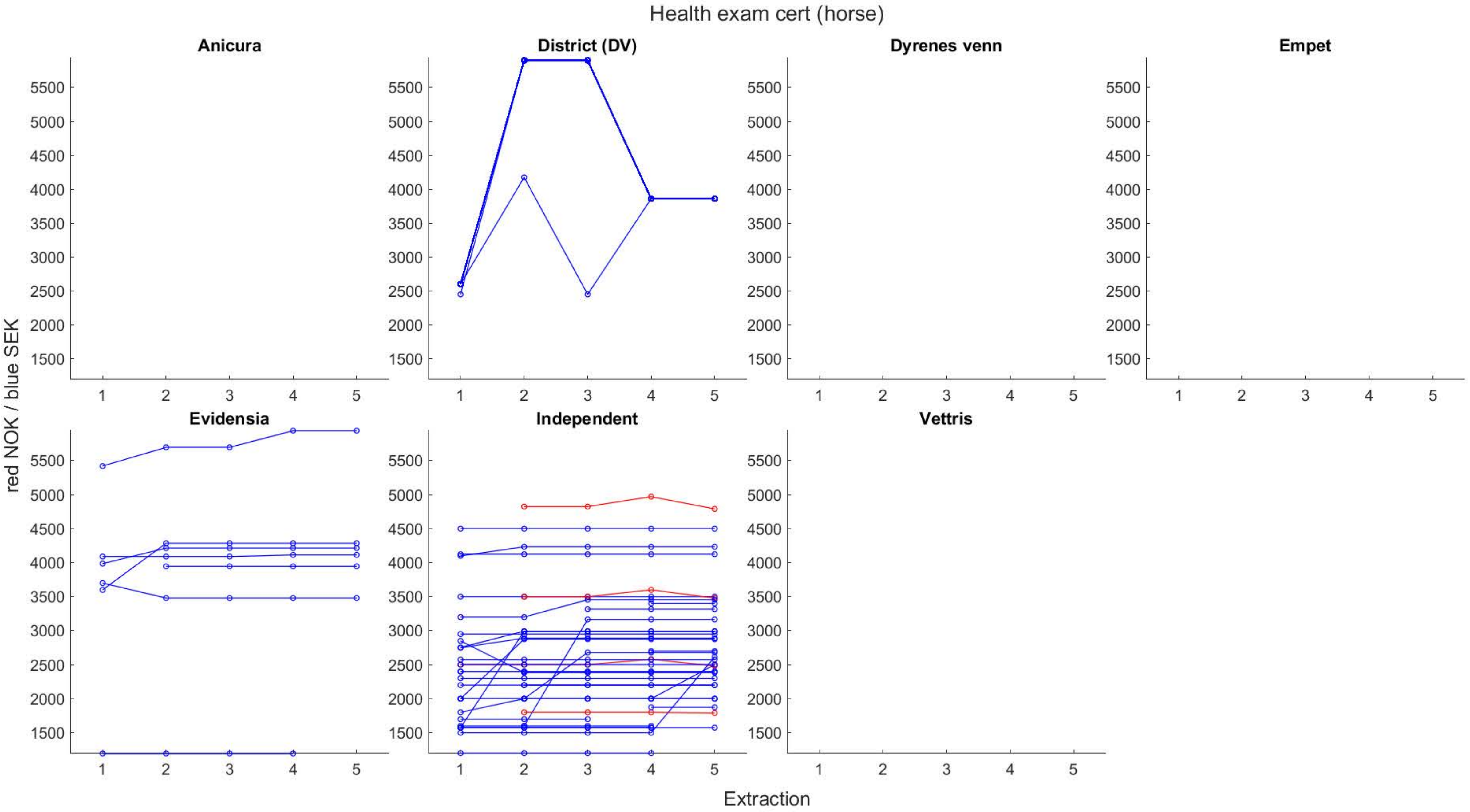

Health exam cert (horse)
Anicura
District (DV)
Dyrenes venn
Empet
Evidensia
Independent
Vettris
red NOK / blue SEK
Extraction
1500
2000
2500
3000
3500
4000
4500
5000
5500
1
2
3
4
5

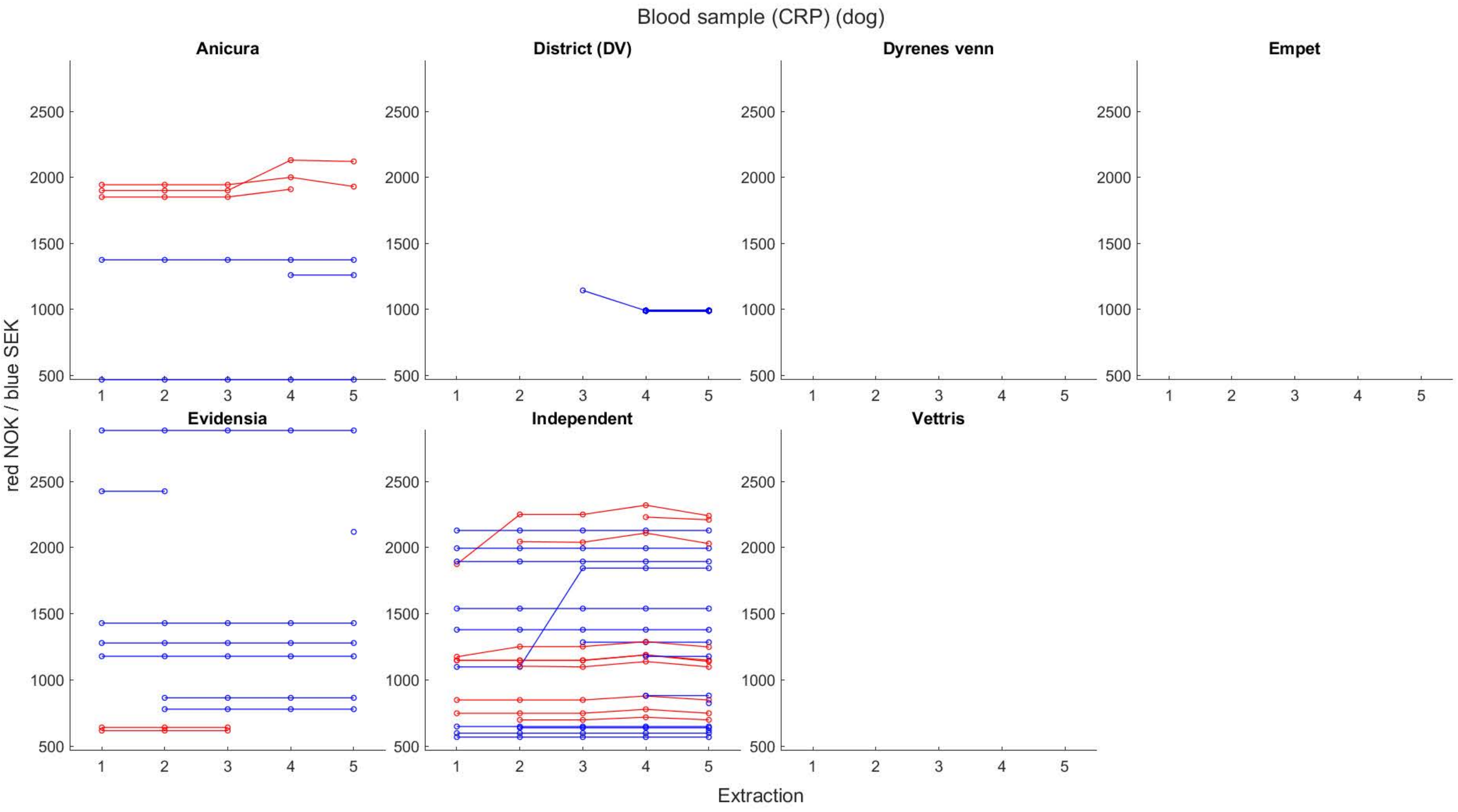

Blood sample (CRP) (dog)
Anicura
District (DV)
Dyrenes venn
Empet
Evidensia
Independent
Vettris
red NOK / blue SEK
Extraction
500
1000
1500
2000
2500
1
2
3
4
5

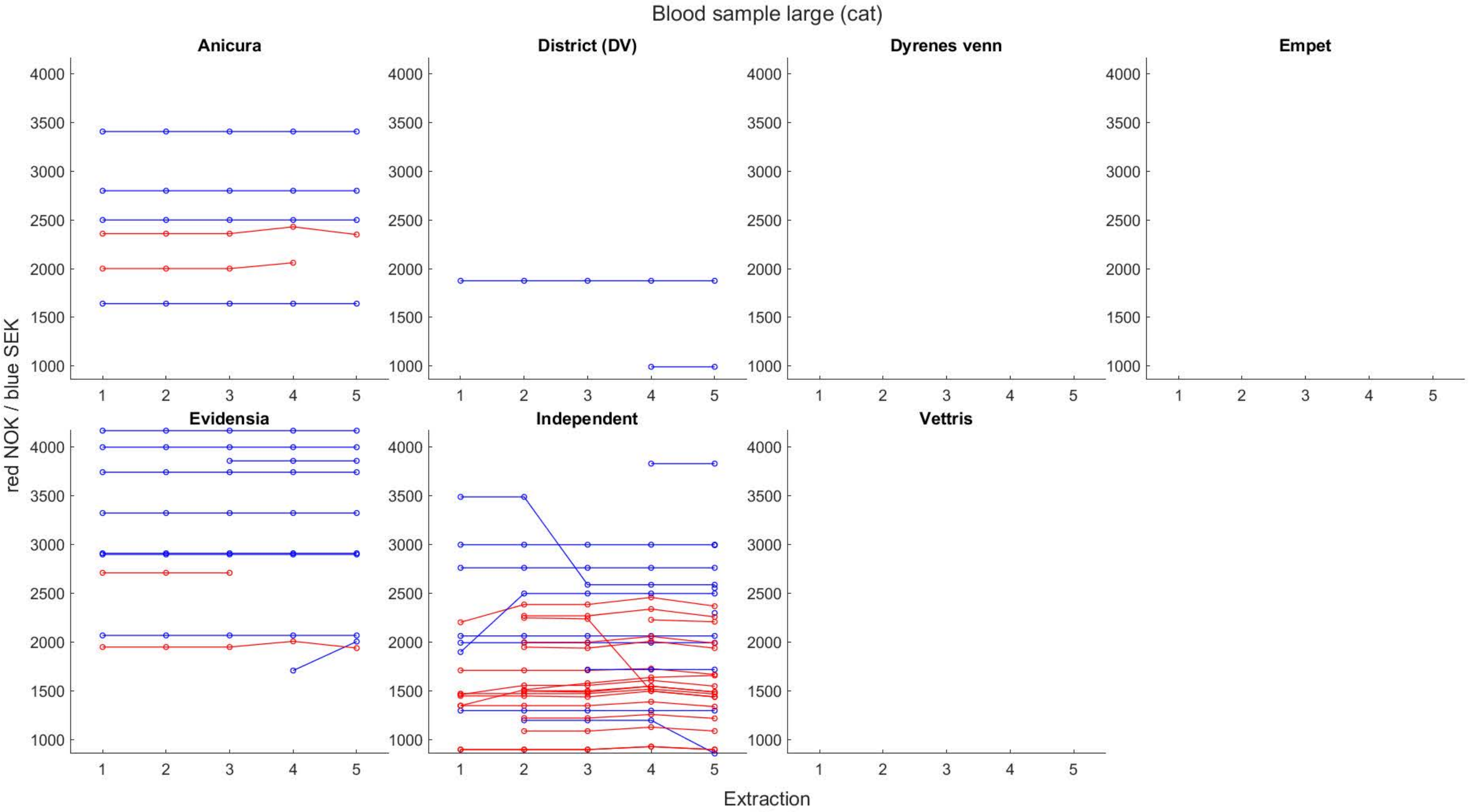

Blood sample large (cat)
Anicura
District (DV)
Dyrenes venn
Empet
Evidensia
Independent
Vettris
red NOK / blue SEK
Extraction

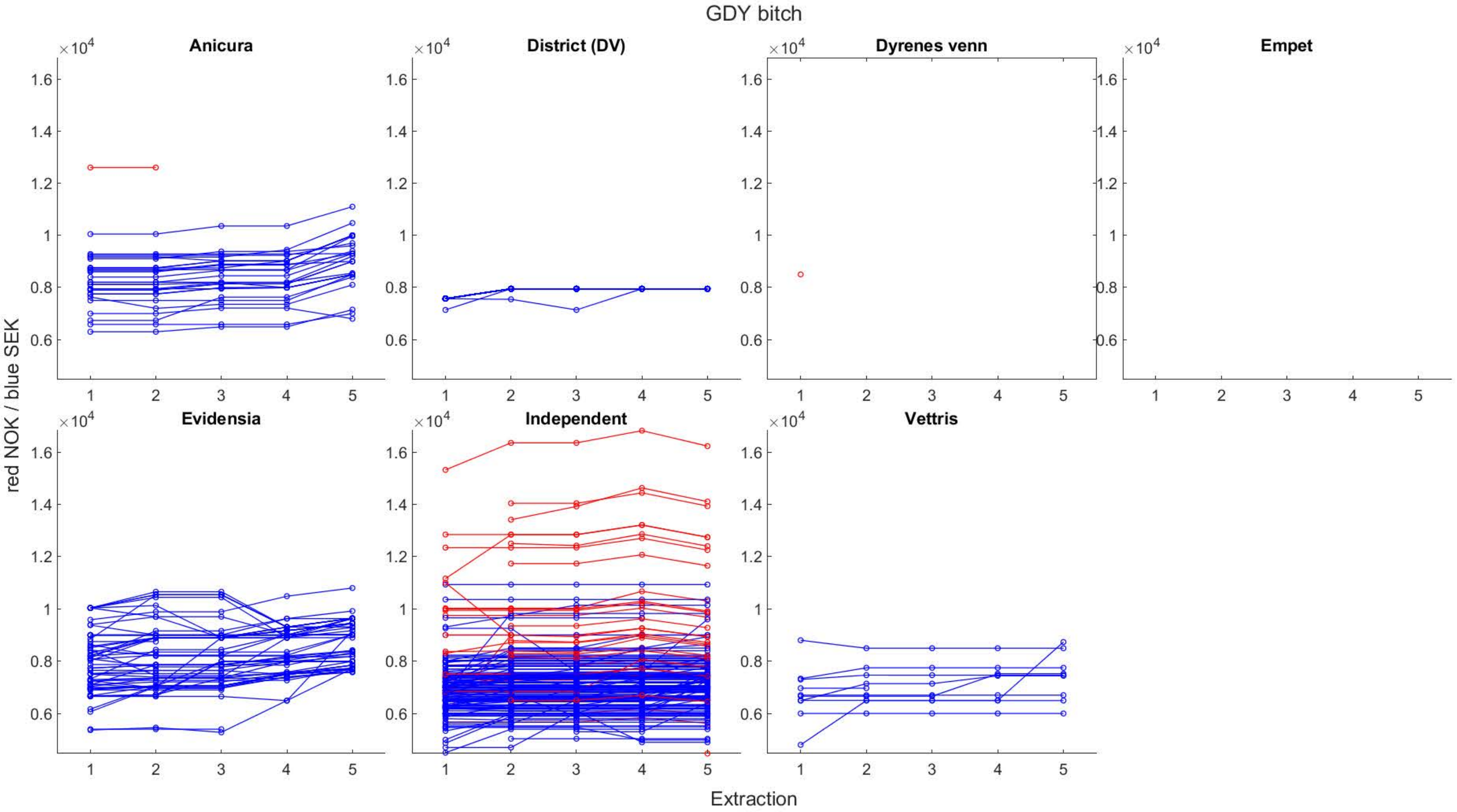
GDY bitch
Anicura
District (DV)
Dyrenes venn
Empet
Evidensia
Independent
Vettris
red NOK / blue SEK
Extraction
×10^4

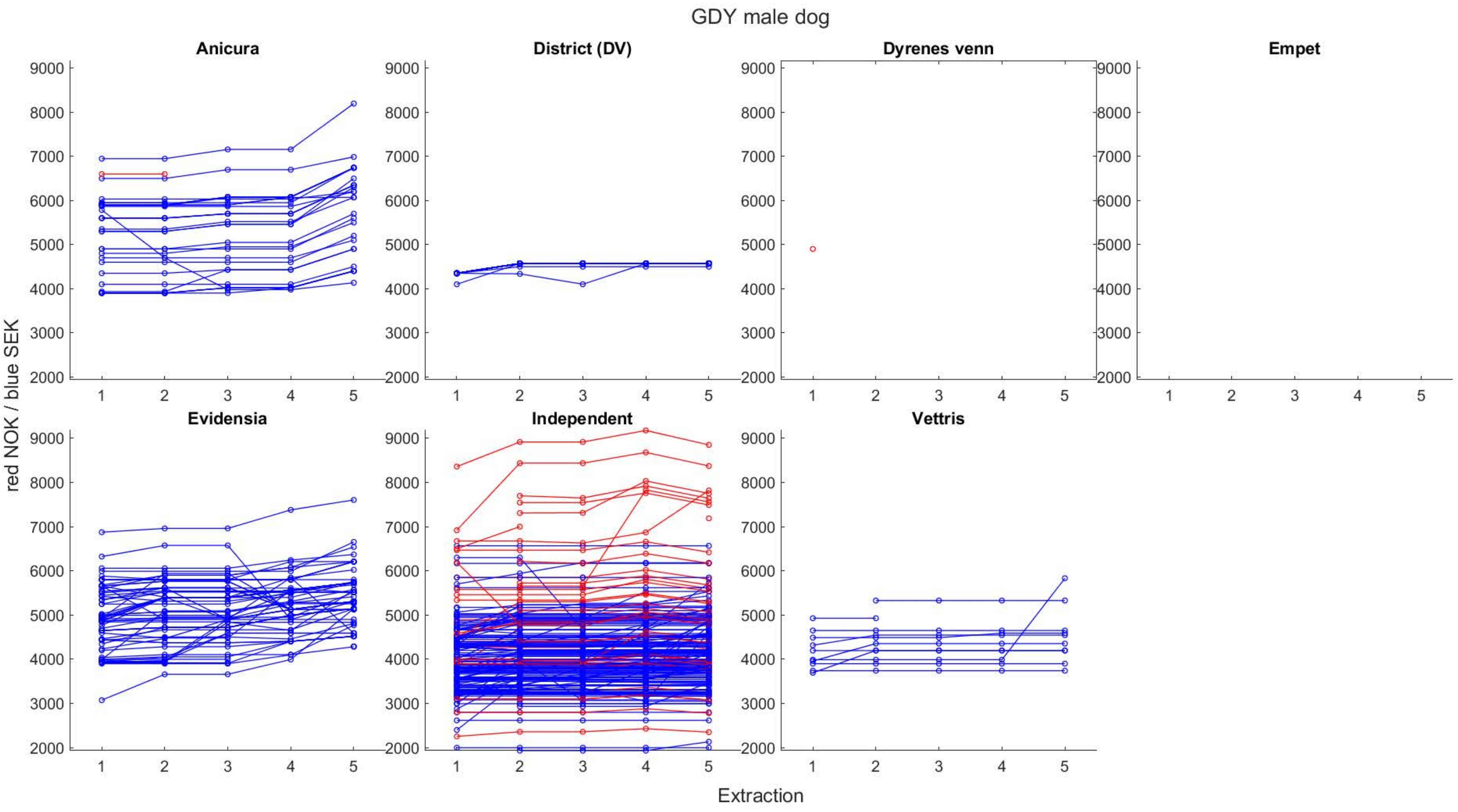
GDY male dog
Anicura
District (DV)
Dyrenes venn
Empet
Evidensia
Independent
Vettris
red NOK / blue SEK
Extraction

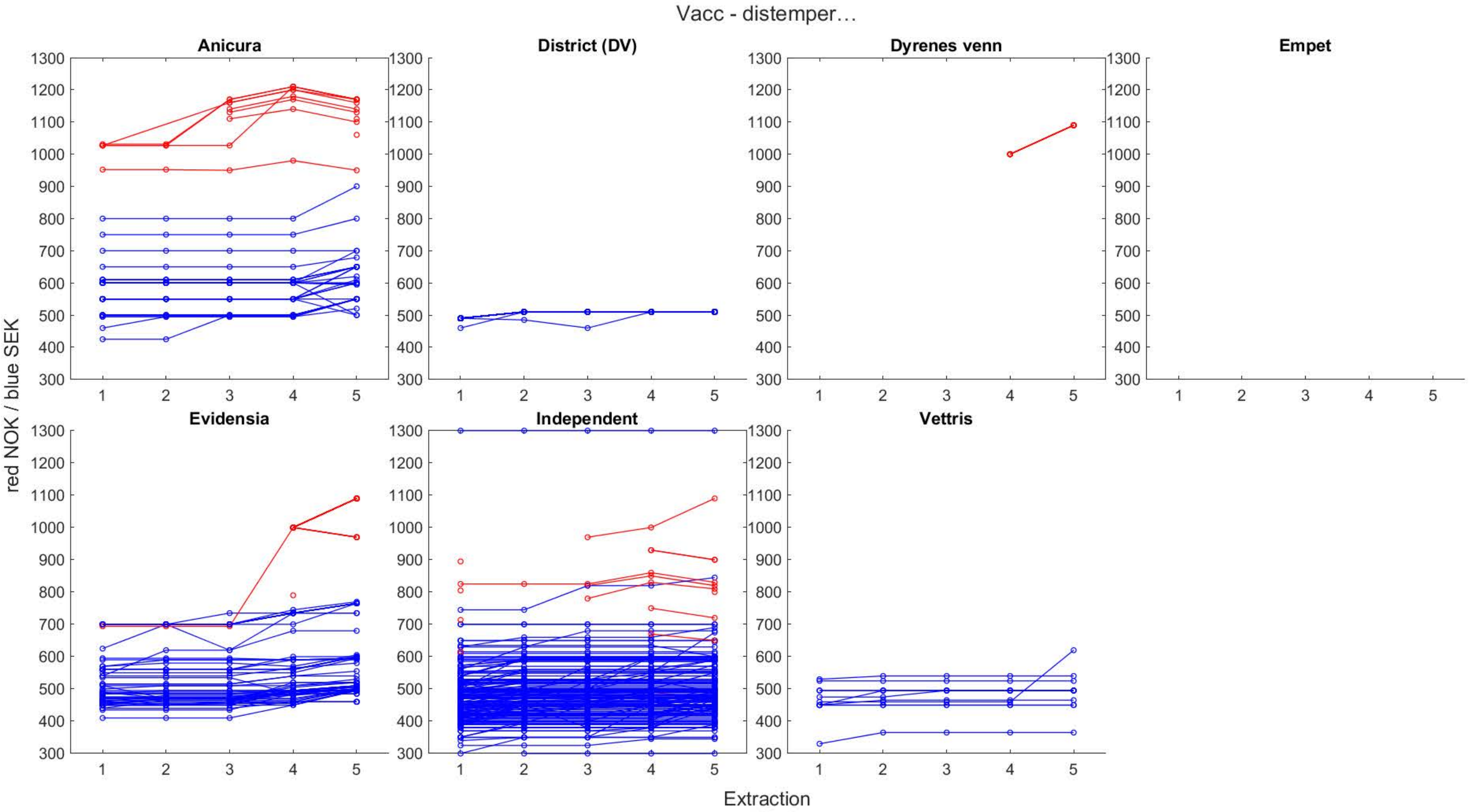

Vacc - distemper...
Anicura
District (DV)
Dyrenes venn
Empet
Evidensia
Independent
Vettris
red NOK / blue SEK
Extraction
1300
1200
1100
1000
900
800
700
600
500
400
300
1
2
3
4
5

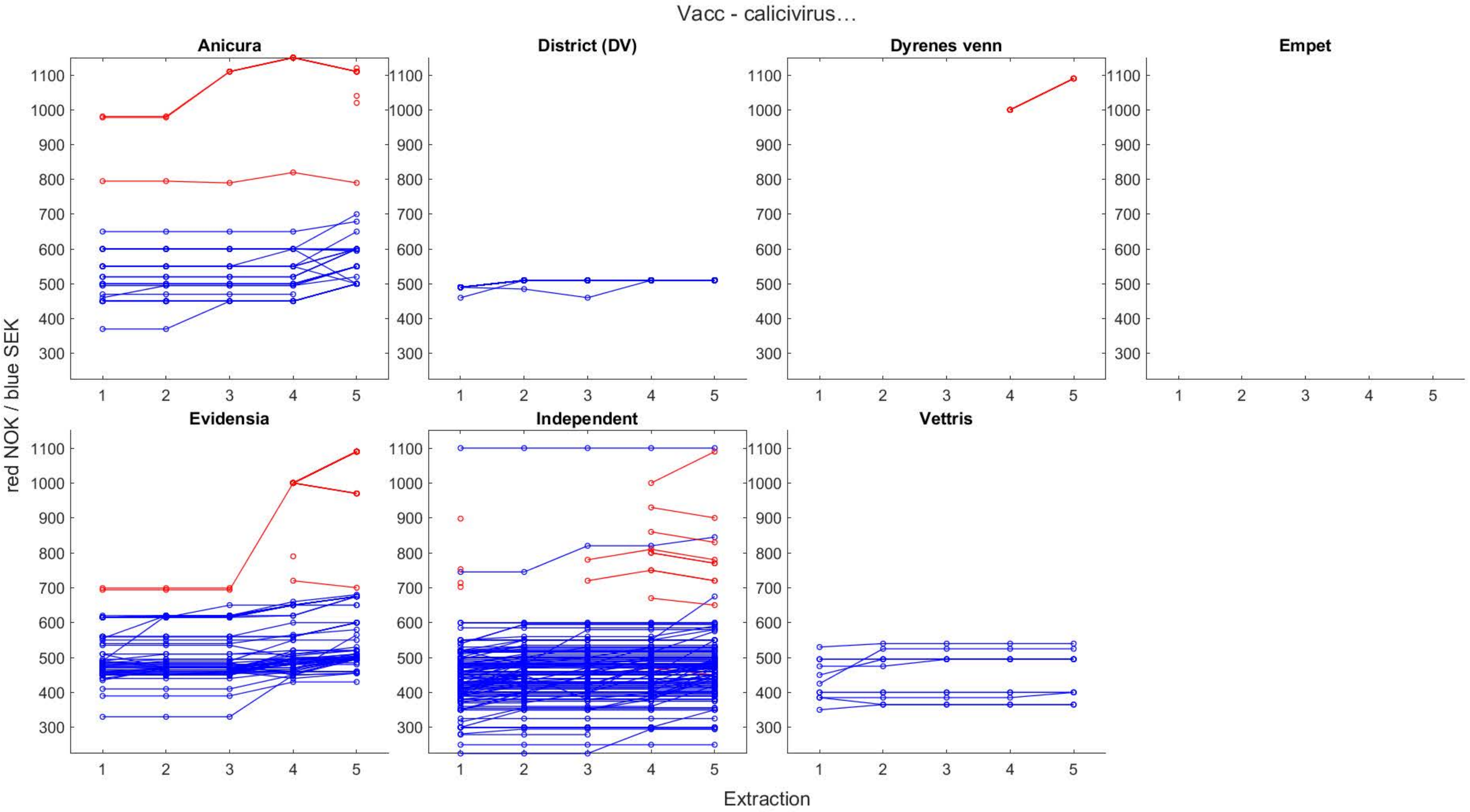
Vacc - calicivirus…
Anicura
District (DV)
Dyrenes venn
Empet
Evidensia
Independent
Vettris
red NOK / blue SEK
Extraction
300
400
500
600
700
800
900
1000
1100
1
2
3
4
5

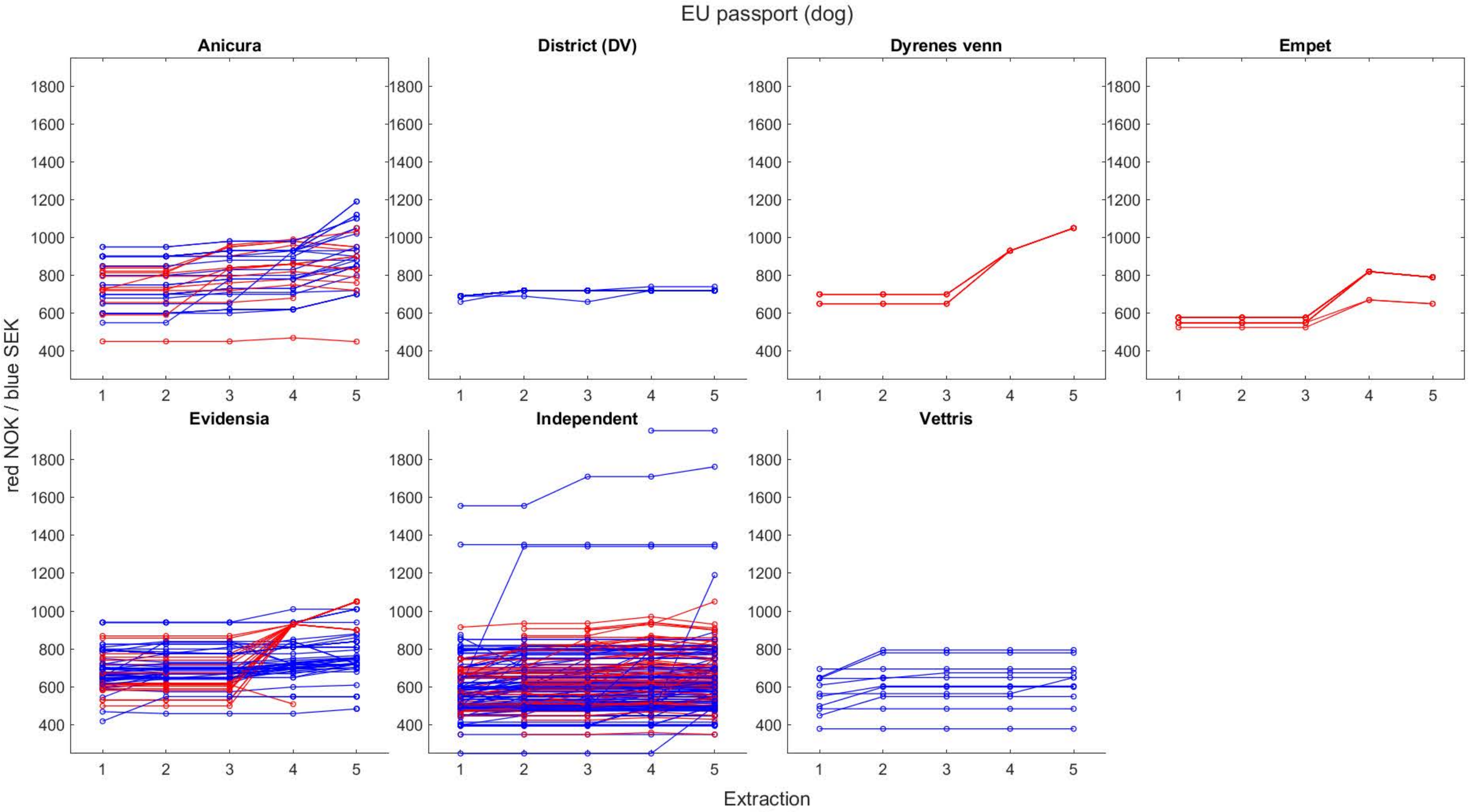

EU passport (dog)
Anicura
District (DV)
Dyrenes venn
Empet
Evidensia
Independent
Vettris
red NOK / blue SEK
Extraction

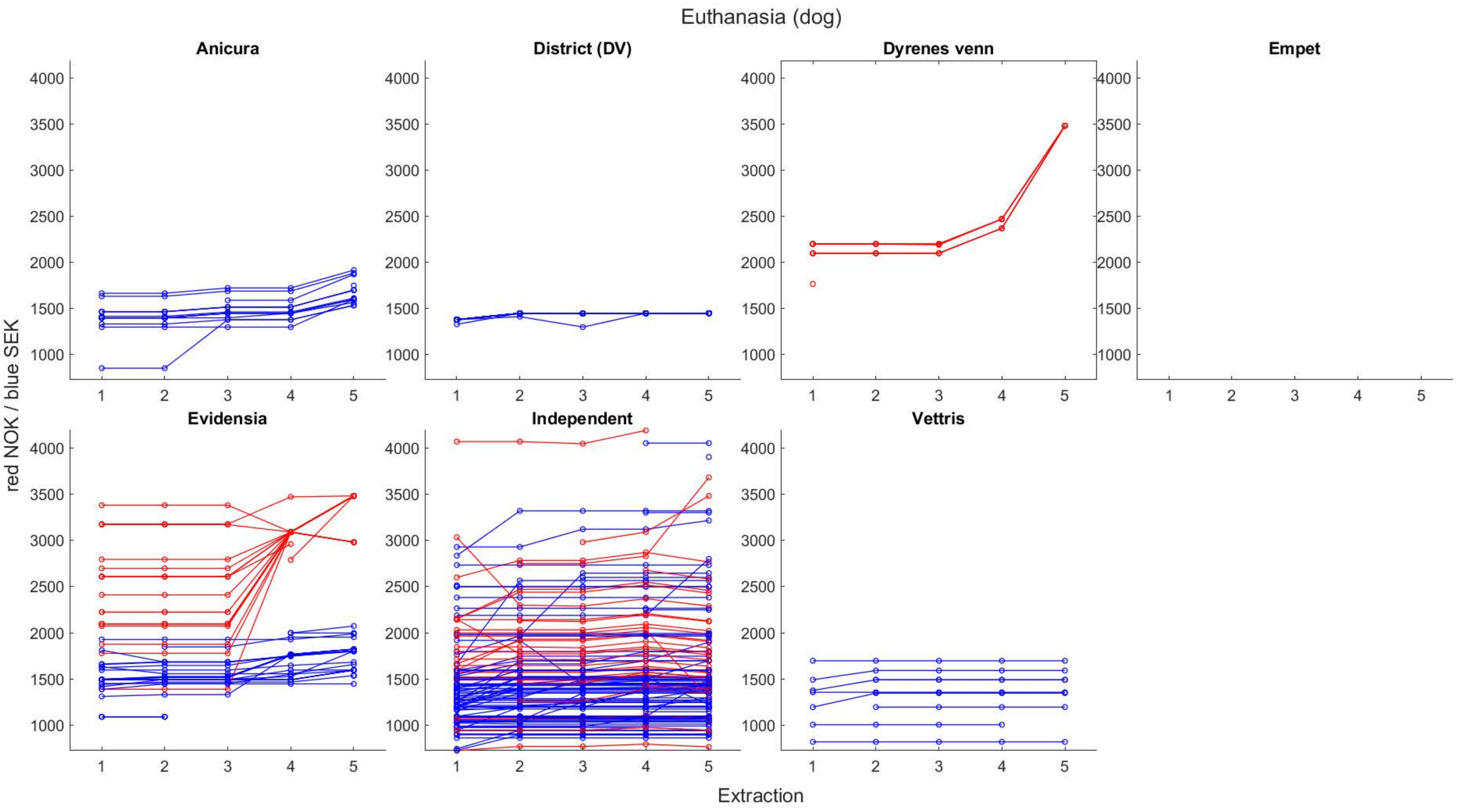
Euthanasia (dog)
Anicura
District (DV)
Dyrenes venn
Empet
Evidensia
Independent
Vettris
red NOK / blue SEK
Extraction

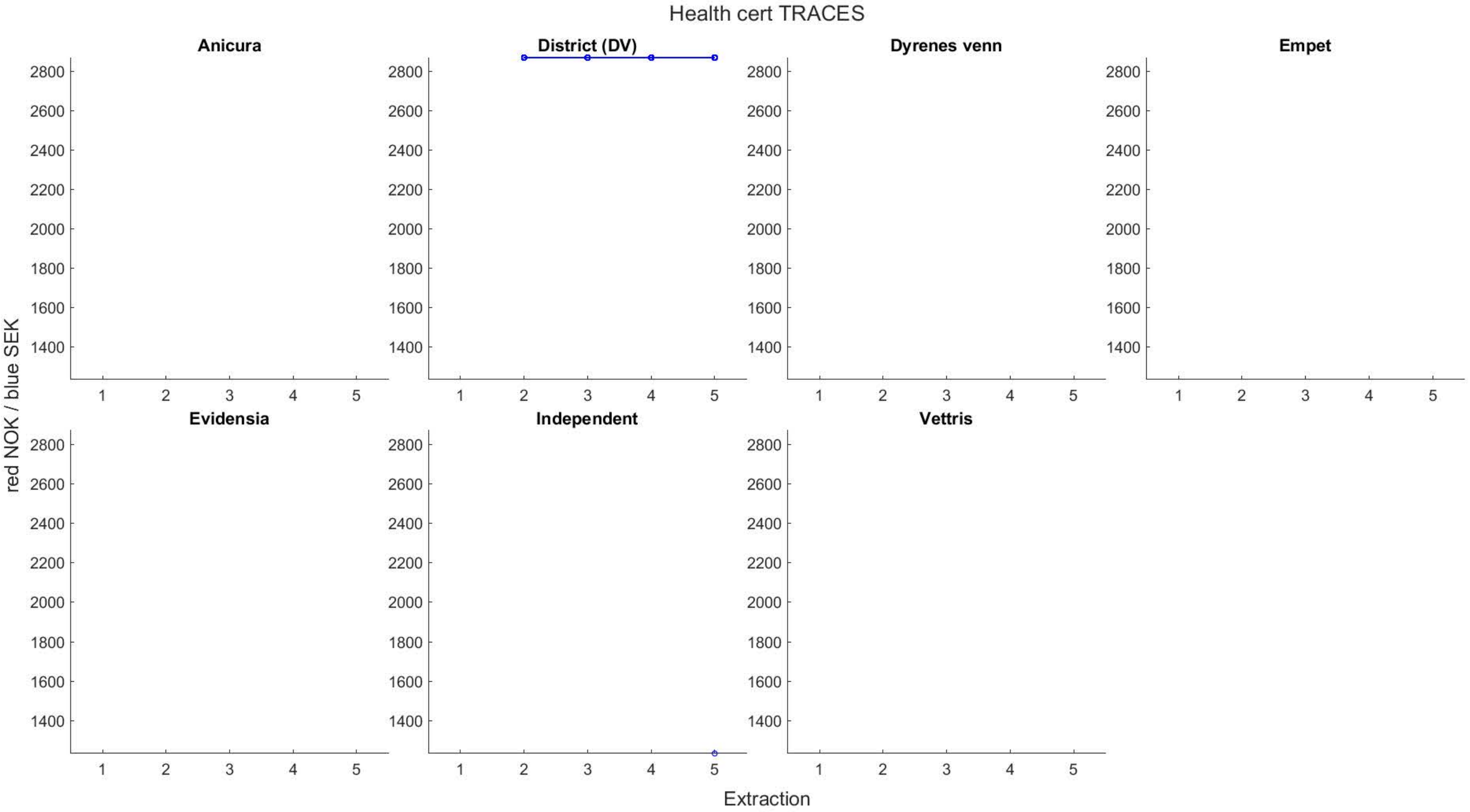

Health cert TRACES
Anicura
District (DV)
Dyrenes venn
Empet
Evidensia
Independent
Vettris
red NOK / blue SEK
Extraction
2800
2600
2400
2200
2000
1800
1600
1400
1
2
3
4
5

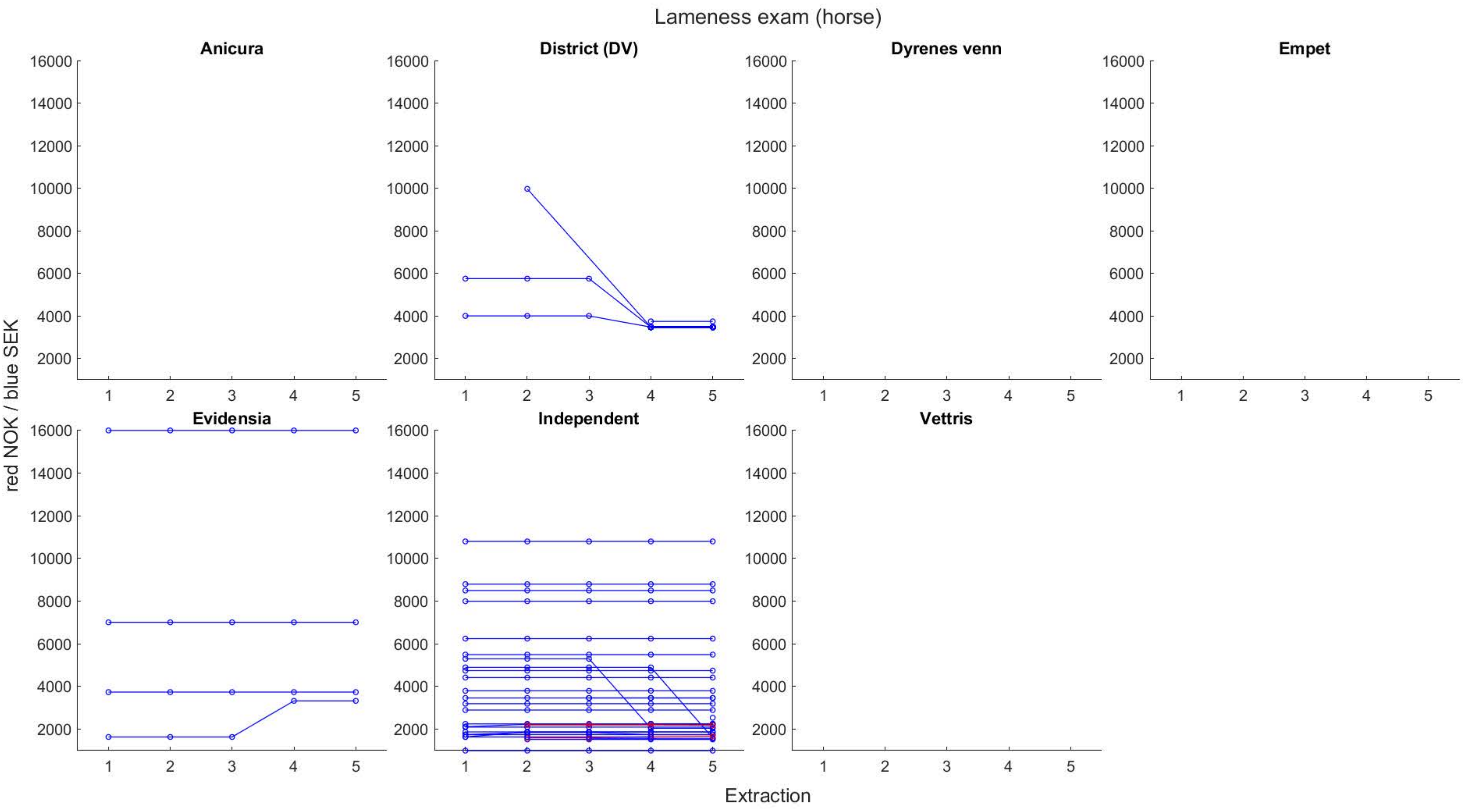

Lameness exam (horse)
Anicura
District (DV)
Dyrenes venn
Empet
Evidensia
Independent
Vettris
red NOK / blue SEK
Extraction

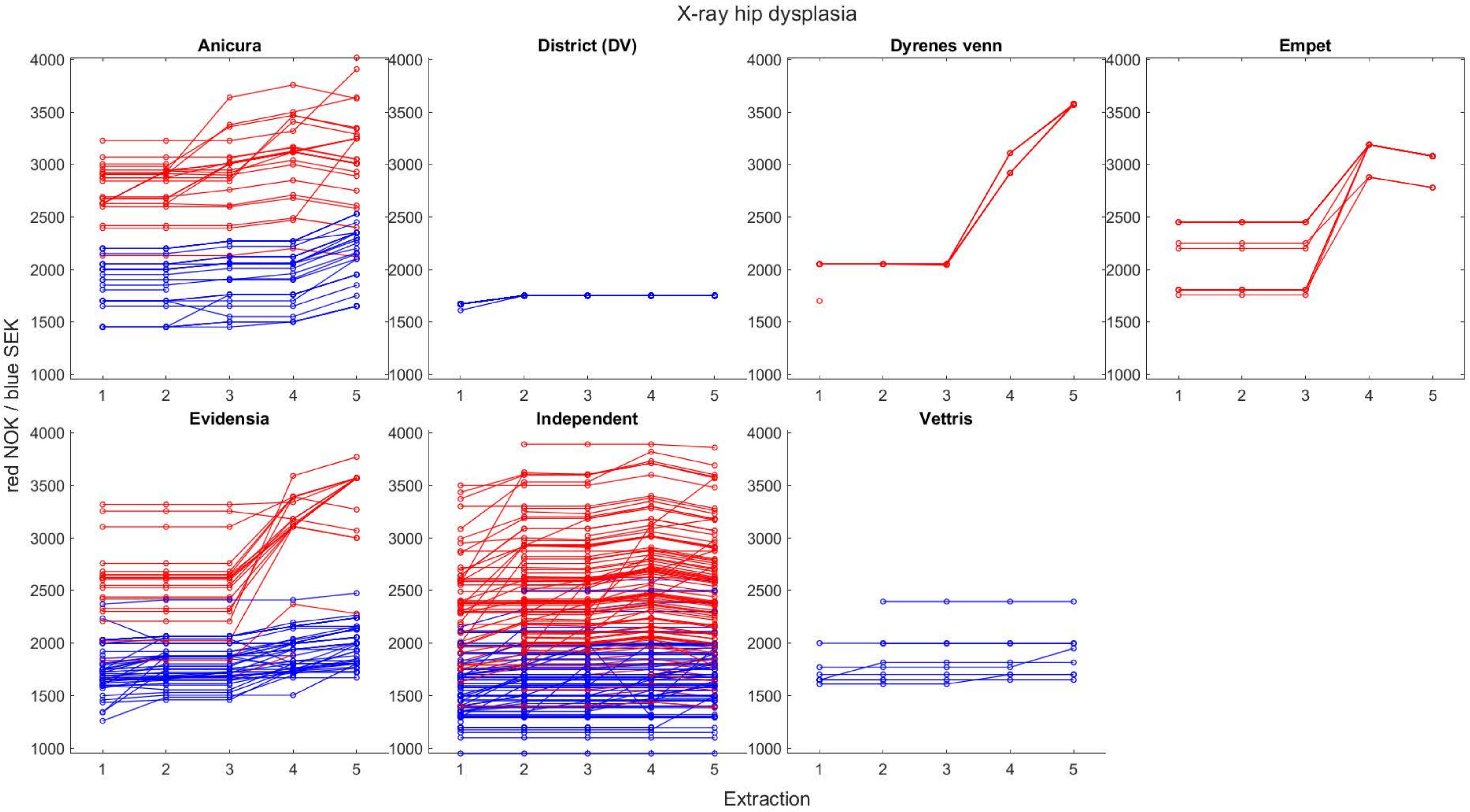

X-ray hip dysplasia
Anicura
District (DV)
Dyrenes venn
Empet
Evidensia
Independent
Vettris
red NOK / blue SEK
Extraction

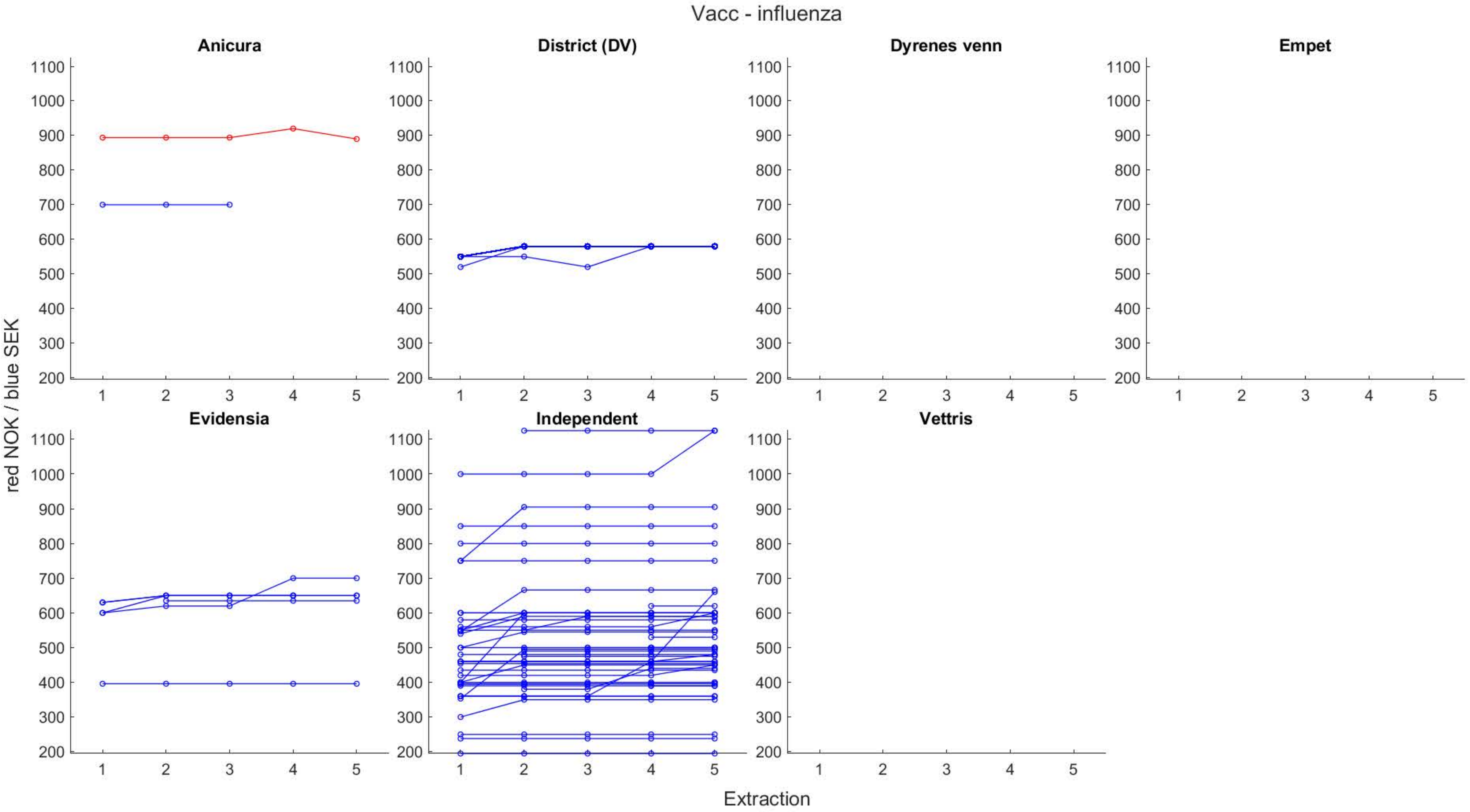

Vacc - influenza
Anicura
District (DV)
Dyrenes venn
Empet
Evidensia
Independent
Vettris
red NOK / blue SEK
Extraction

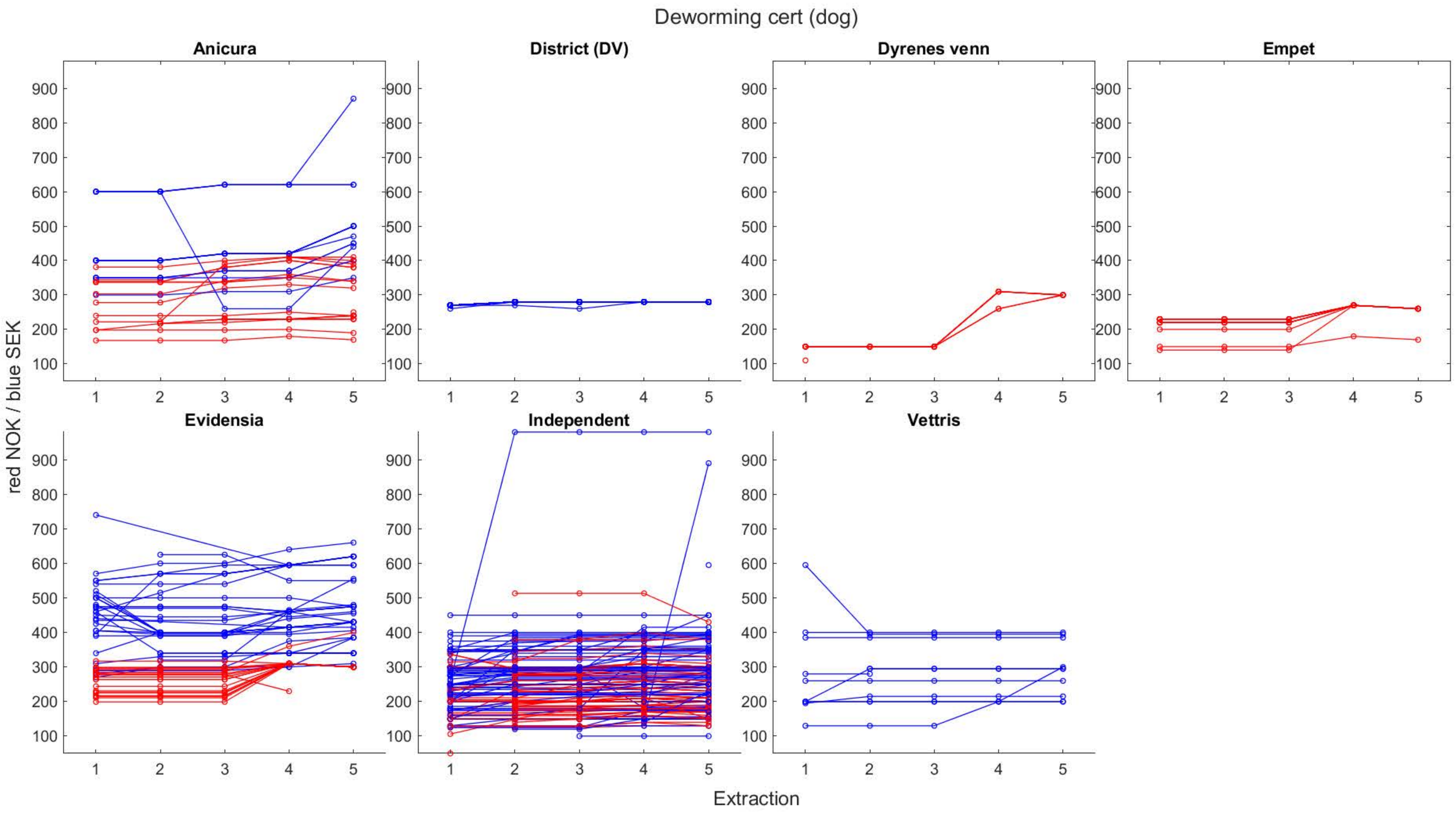

Deworming cert (dog)
Anicura
District (DV)
Dyrenes venn
Empet
Evidensia
Independent
Vettris
red NOK / blue SEK
Extraction
100
200
300
400
500
600
700
800
900
1
2
3
4
5

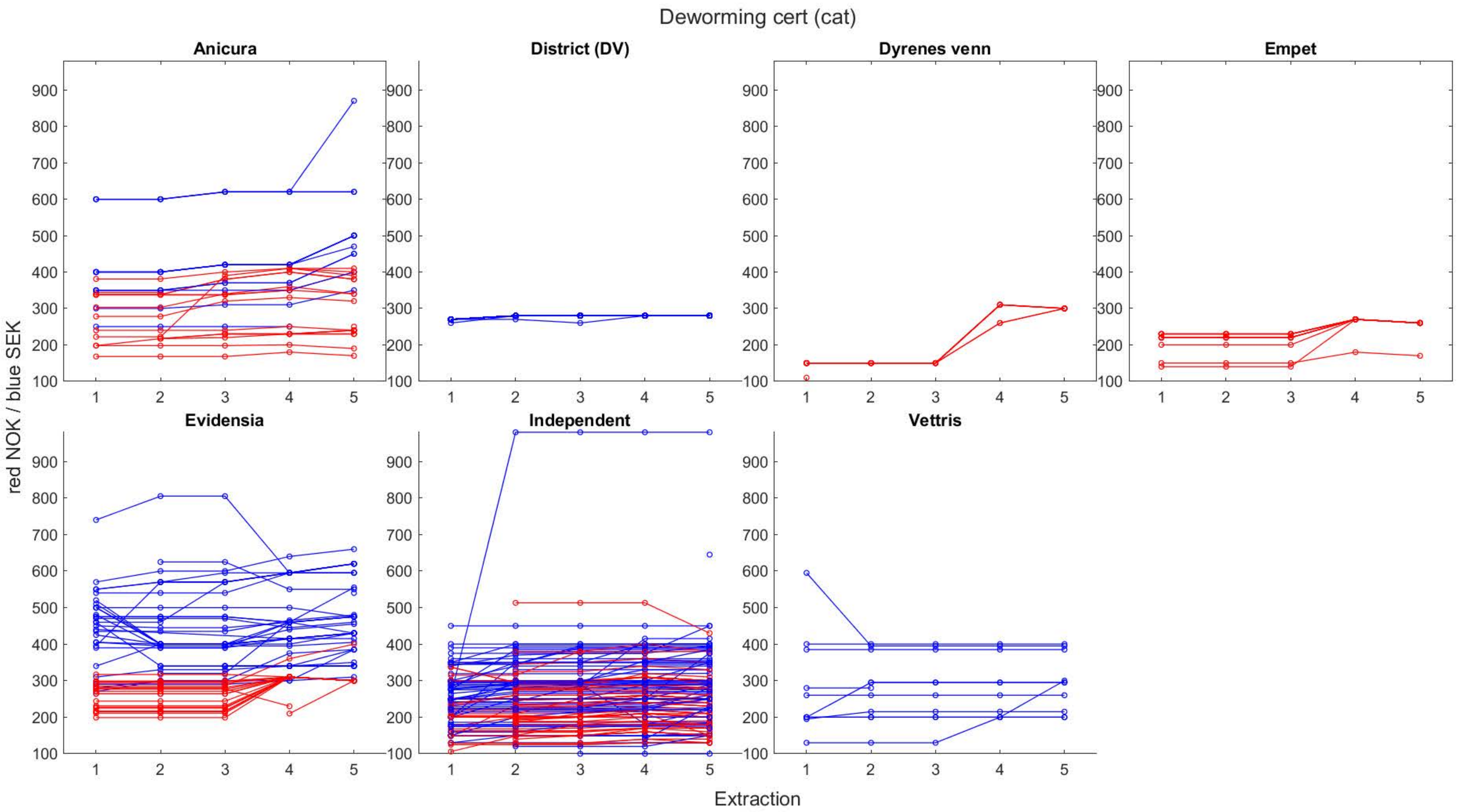

Deworming cert (cat)
Anicura
District (DV)
Dyrenes venn
Empet
Evidensia
Independent
Vettris
red NOK / blue SEK
Extraction

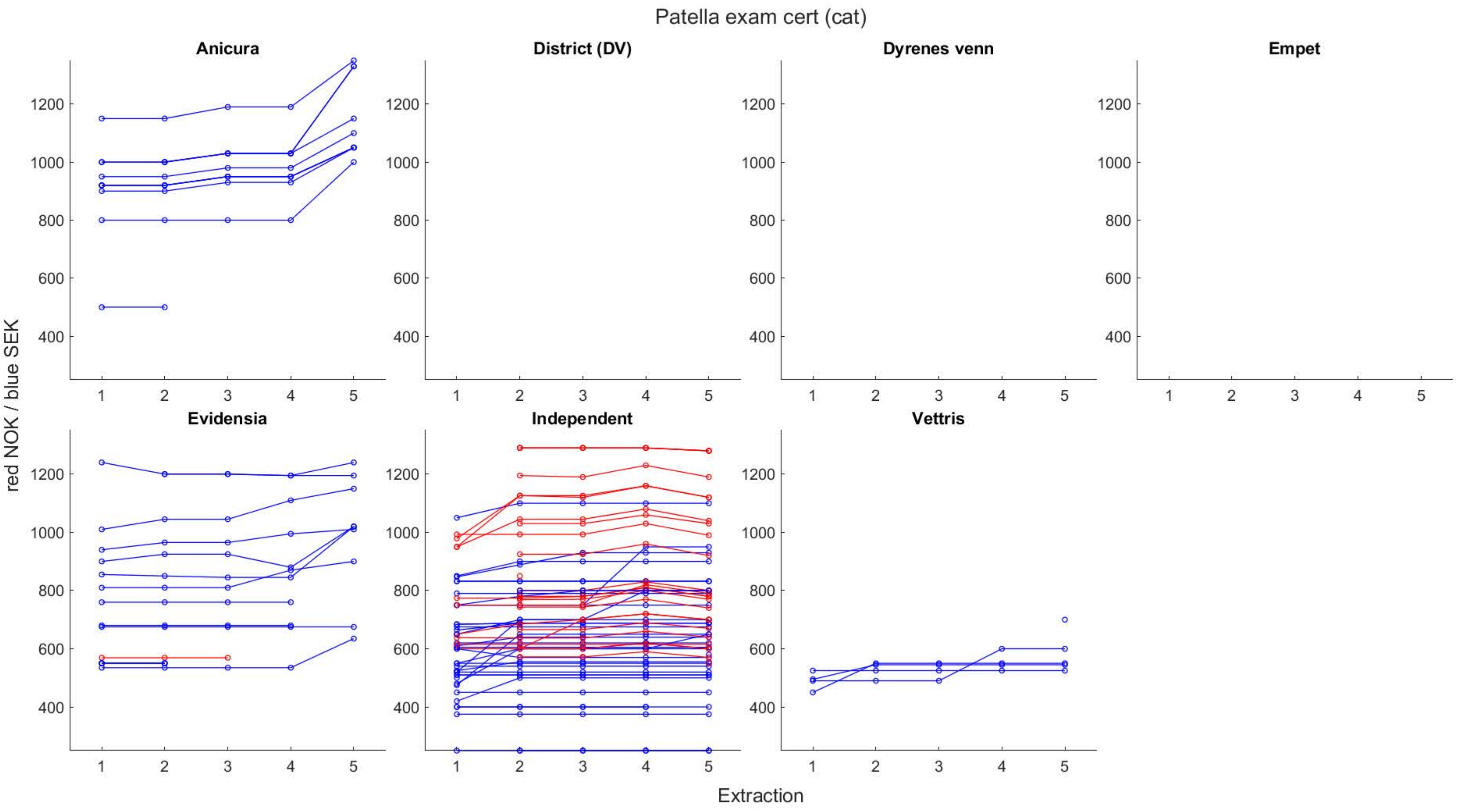
Patella exam cert (cat)
Anicura
District (DV)
Dyrenes venn
Empet
Evidensia
Independent
Vettris
red NOK / blue SEK
Extraction
400
600
800
1000
1200
1
2
3
4
5

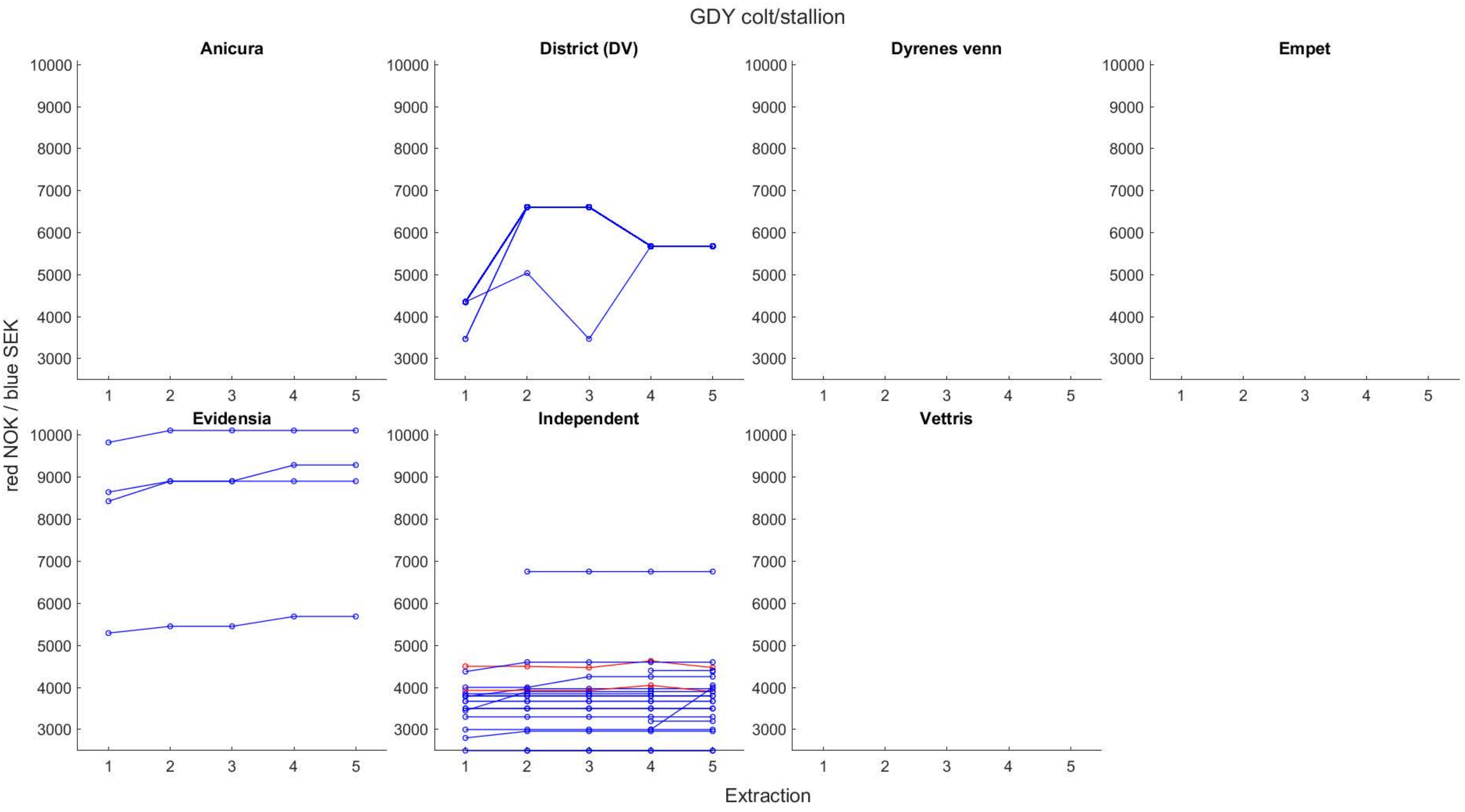

GDY colt/stallion
Anicura
District (DV)
Dyrenes venn
Empet
Evidensia
Independent
Vettris
red NOK / blue SEK
Extraction
10000
9000
8000
7000
6000
5000
4000
3000
1
2
3
4
5

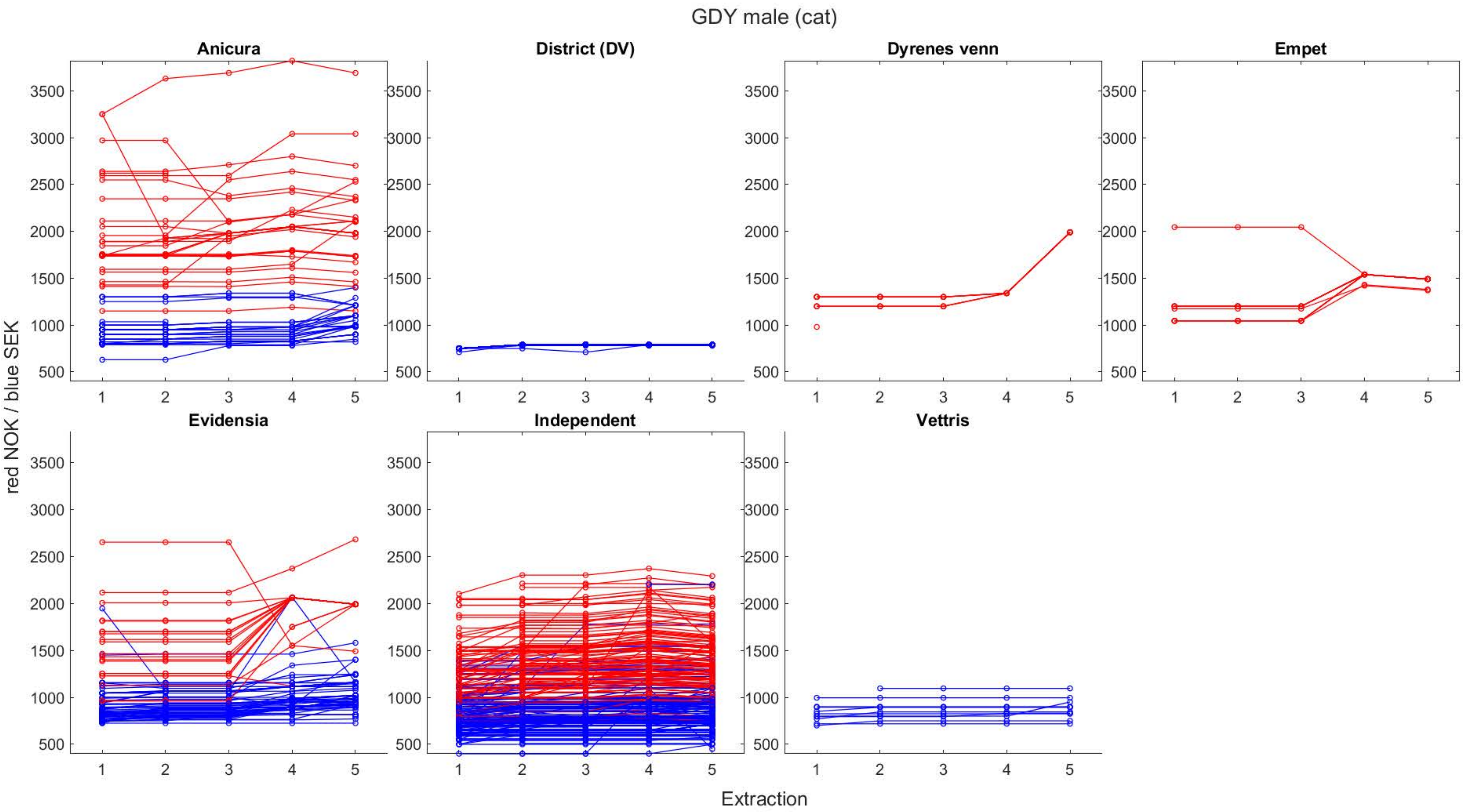

GDY male (cat)
Anicura
District (DV)
Dyrenes venn
Empet
Evidensia
Independent
Vettris
red NOK / blue SEK
Extraction

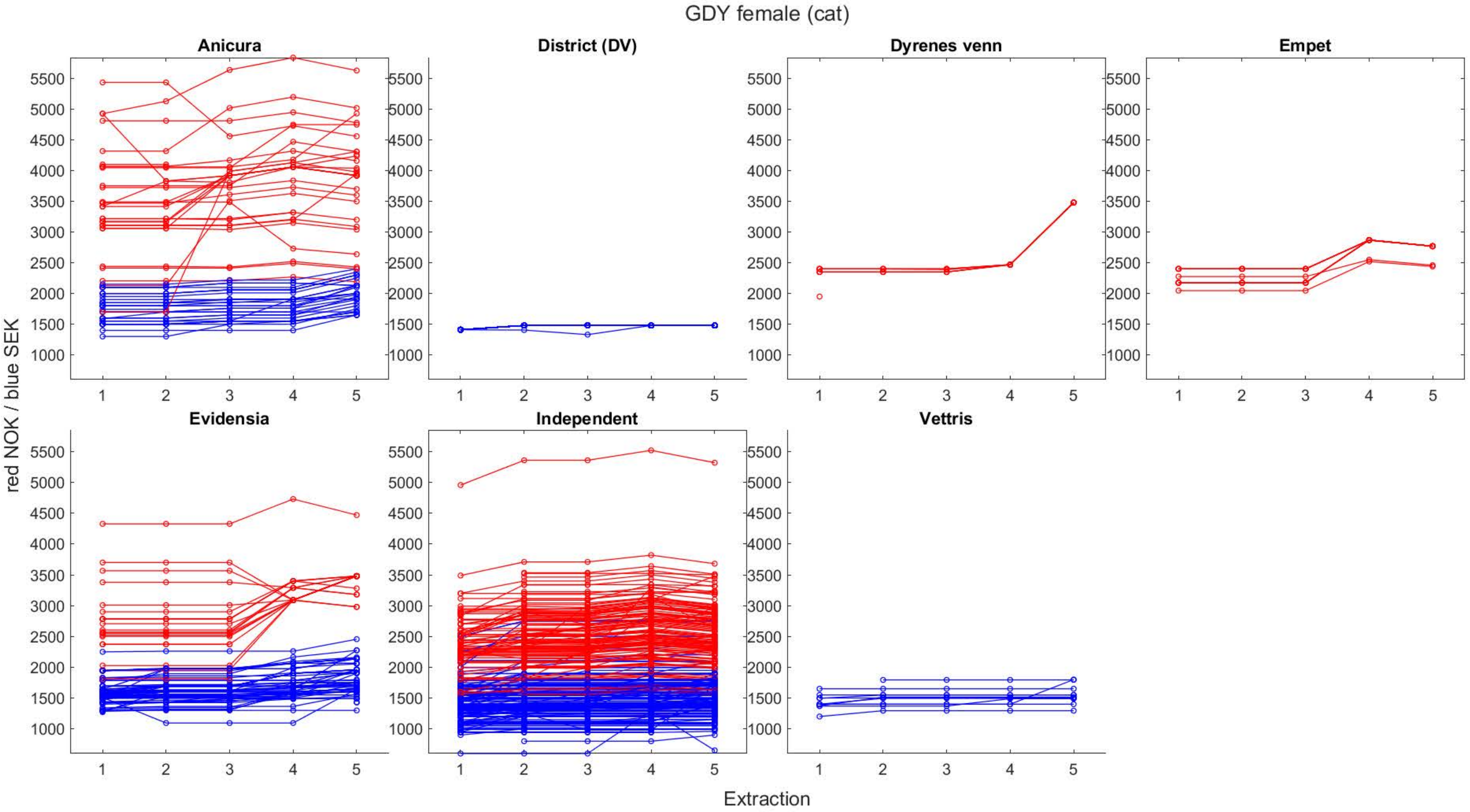

GDY female (cat)
Anicura
District (DV)
Dyrenes venn
Empet
Evidensia
Independent
Vettris
red NOK / blue SEK
Extraction

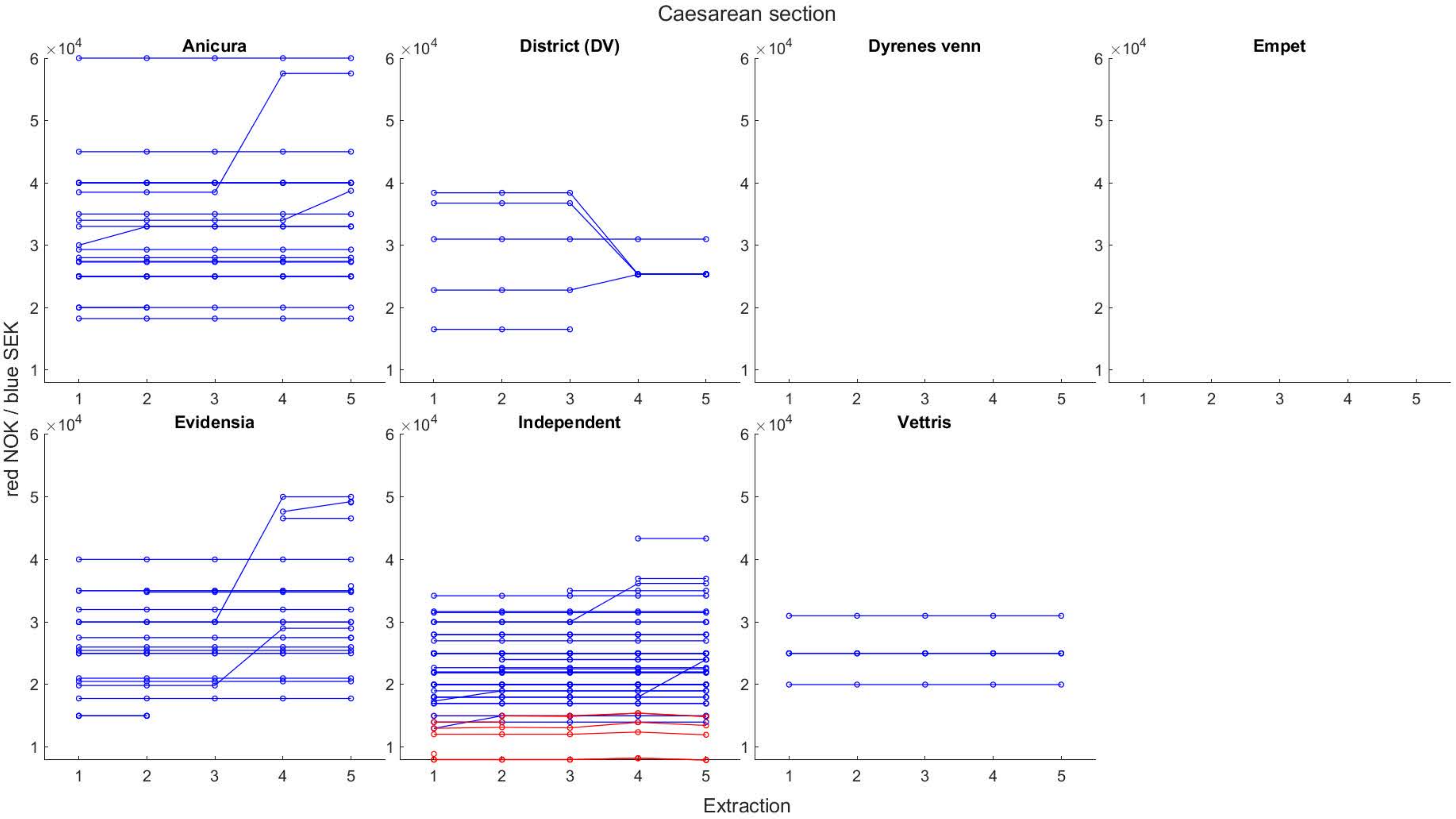

Caesarean section
Anicura
District (DV)
Dyrenes venn
Empet
Evidensia
Independent
Vettris
red NOK / blue SEK
Extraction
×10^4

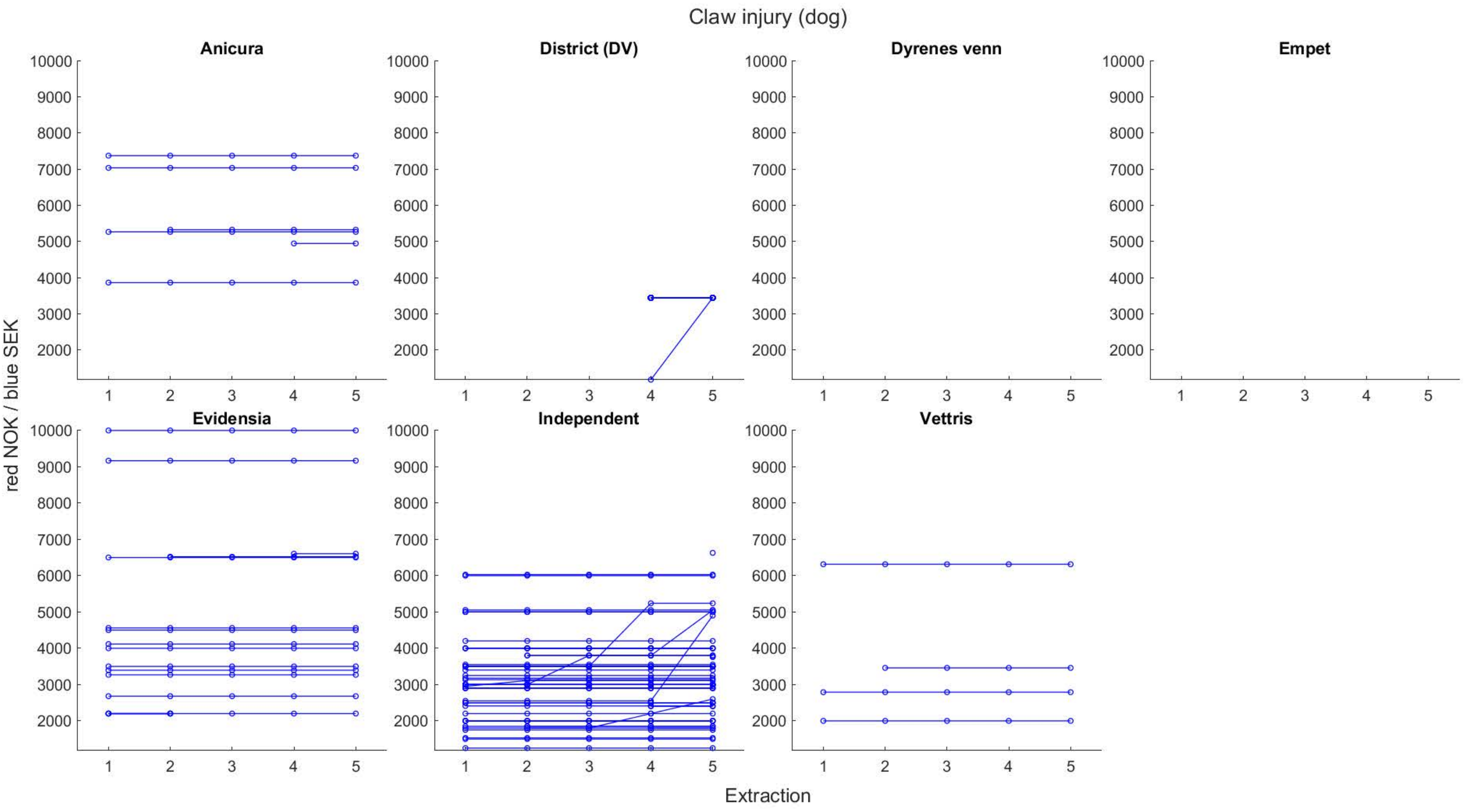

Claw injury (dog)
Anicura
District (DV)
Dyrenes venn
Empet
Evidensia
Independent
Vettris
red NOK / blue SEK
Extraction

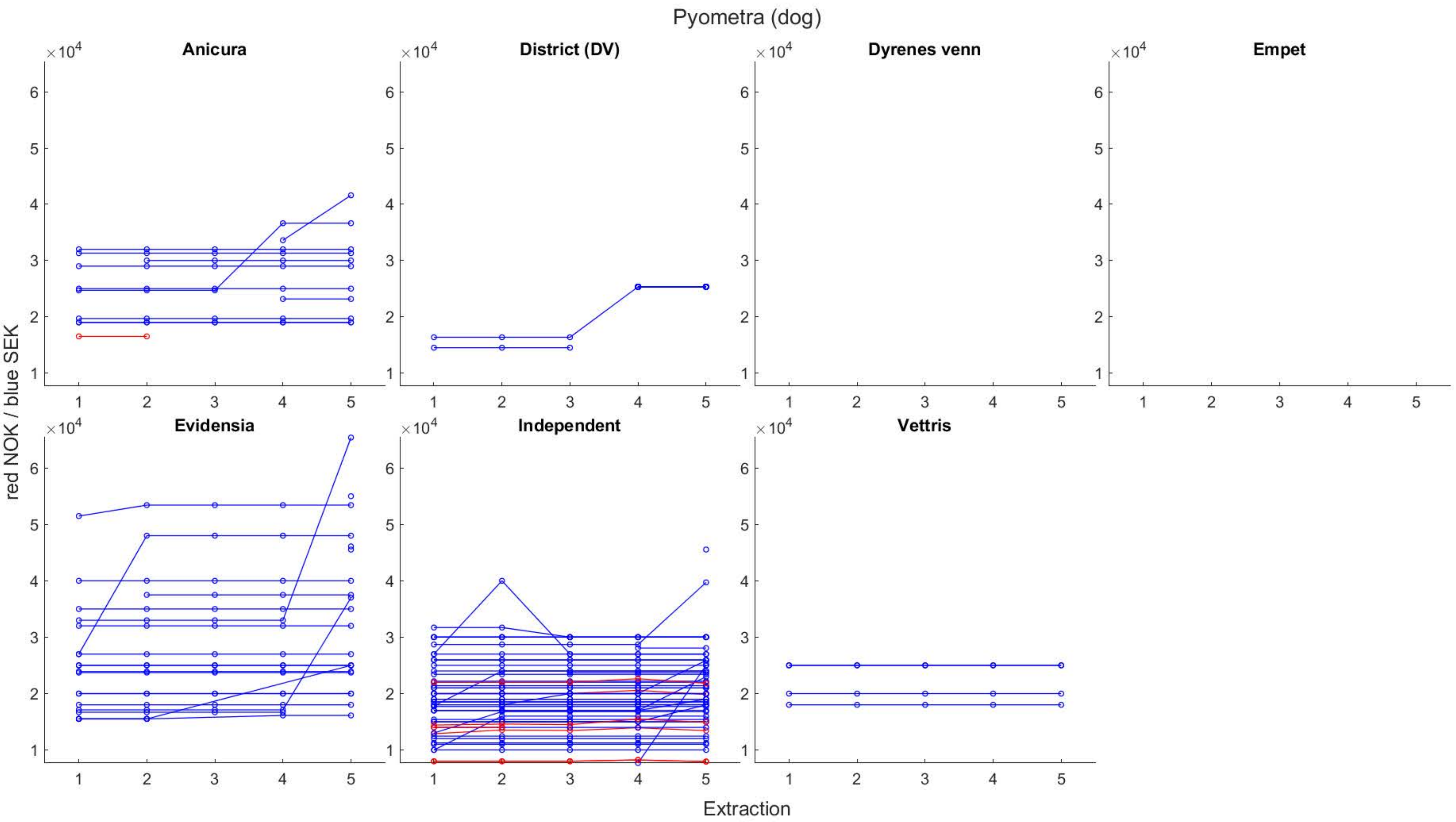

Pyometra (dog)
Anicura
District (DV)
Dyrenes venn
Empet
Evidensia
Independent
Vettris
×10^4
red NOK / blue SEK
Extraction

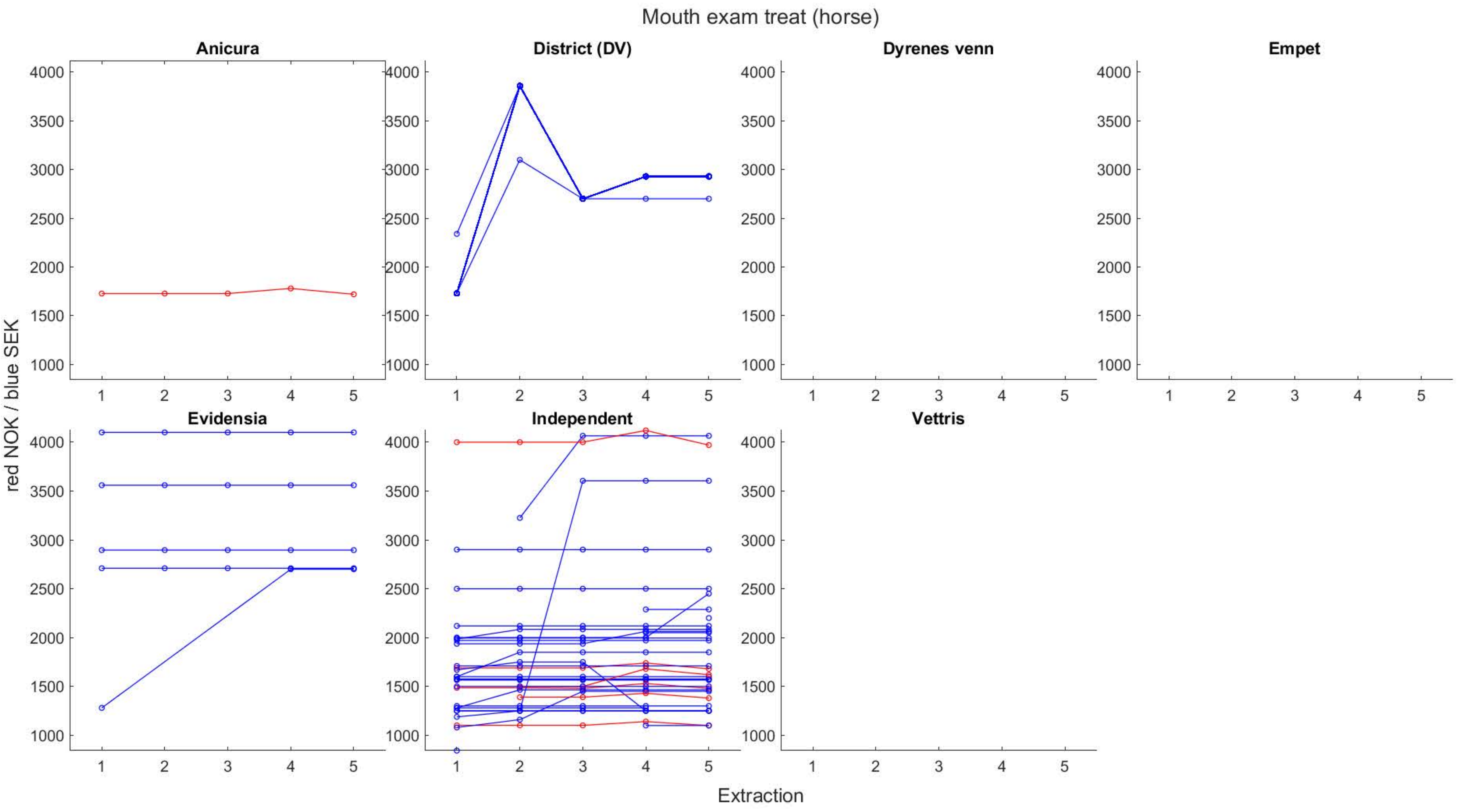
Mouth exam treat (horse)
Anicura
District (DV)
Dyrenes venn
Empet
Evidensia
Independent
Vettris
red NOK / blue SEK
Extraction

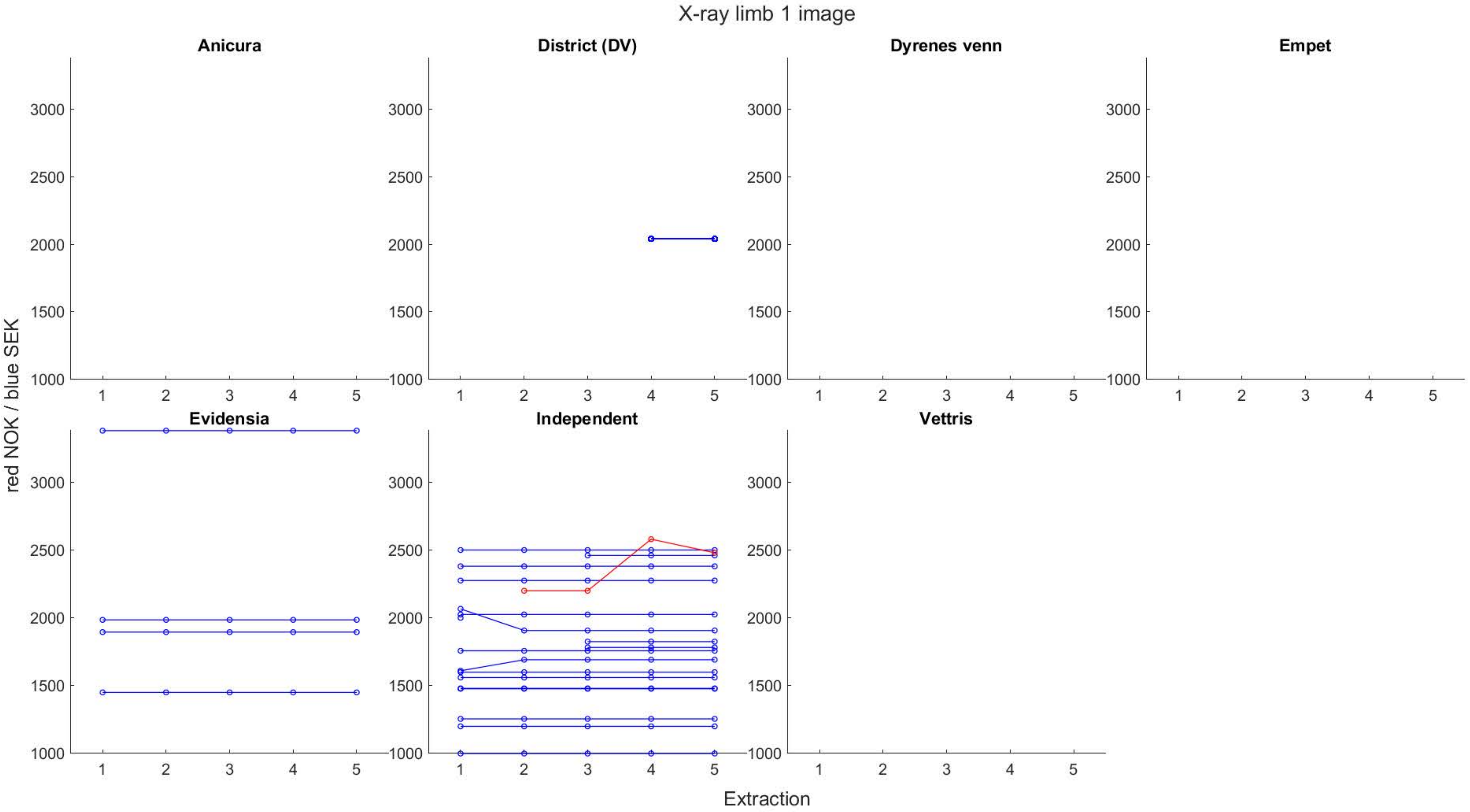
X-ray limb 1 image
Anicura
District (DV)
Dyrenes venn
Empet
Evidensia
Independent
Vettris
red NOK / blue SEK
Extraction
1000
1500
2000
2500
3000
1
2
3
4
5

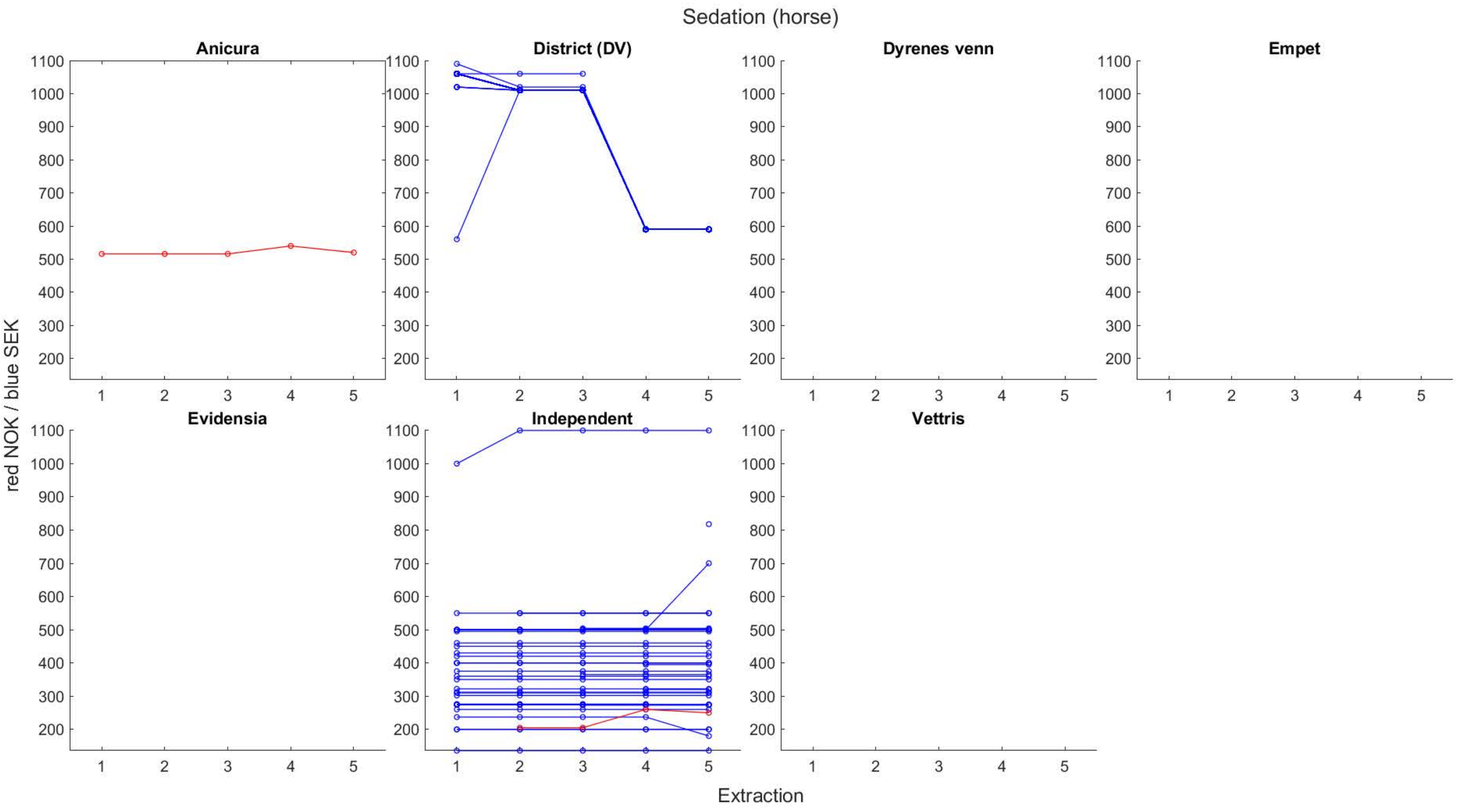

Sedation (horse)
Anicura
District (DV)
Dyrenes venn
Empet
Evidensia
Independent
Vettris
red NOK / blue SEK
Extraction

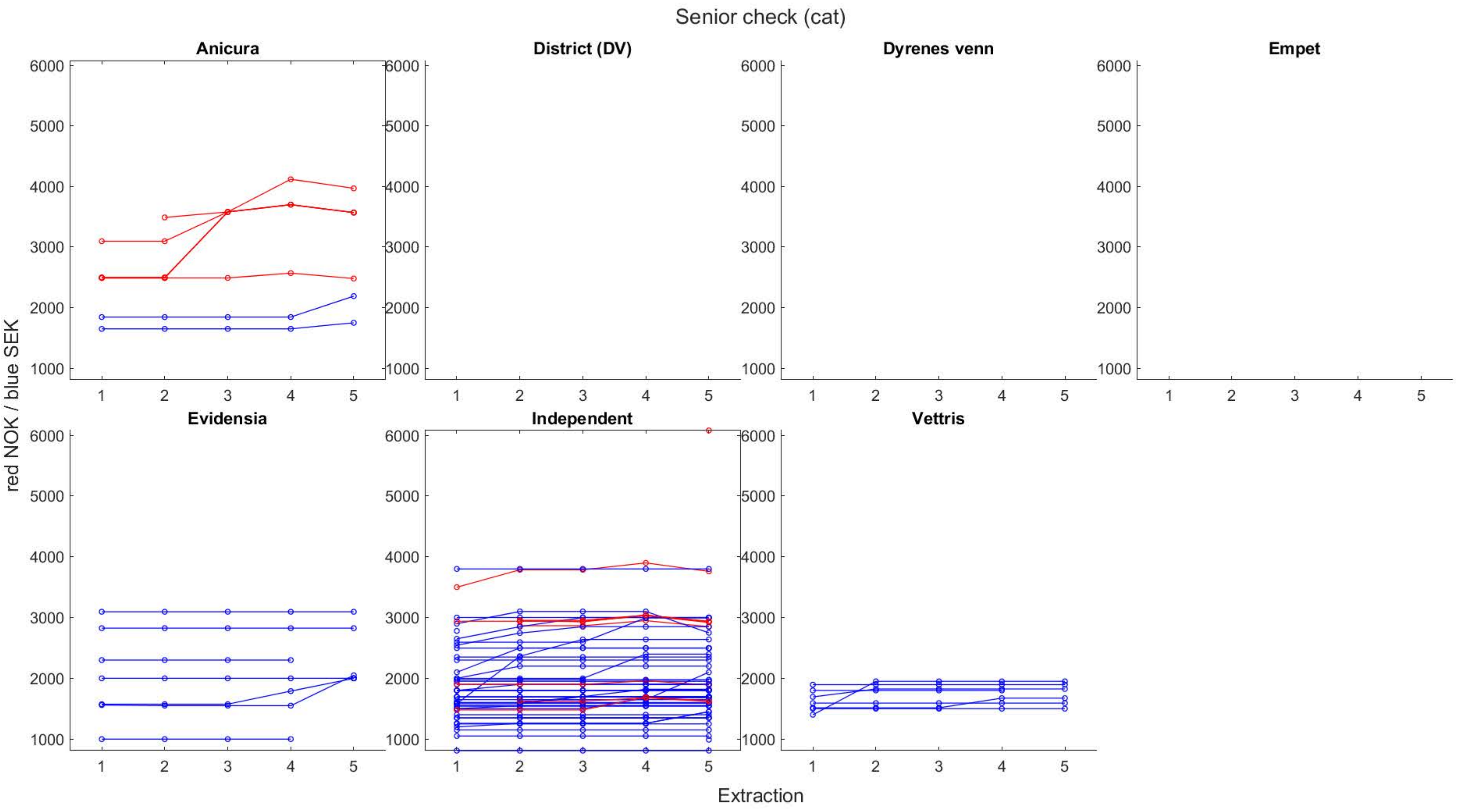

Senior check (cat)
Anicura
District (DV)
Dyrenes venn
Empet
Evidensia
Independent
Vettris
red NOK / blue SEK
Extraction
1000
2000
3000
4000
5000
6000
1
2
3
4
5

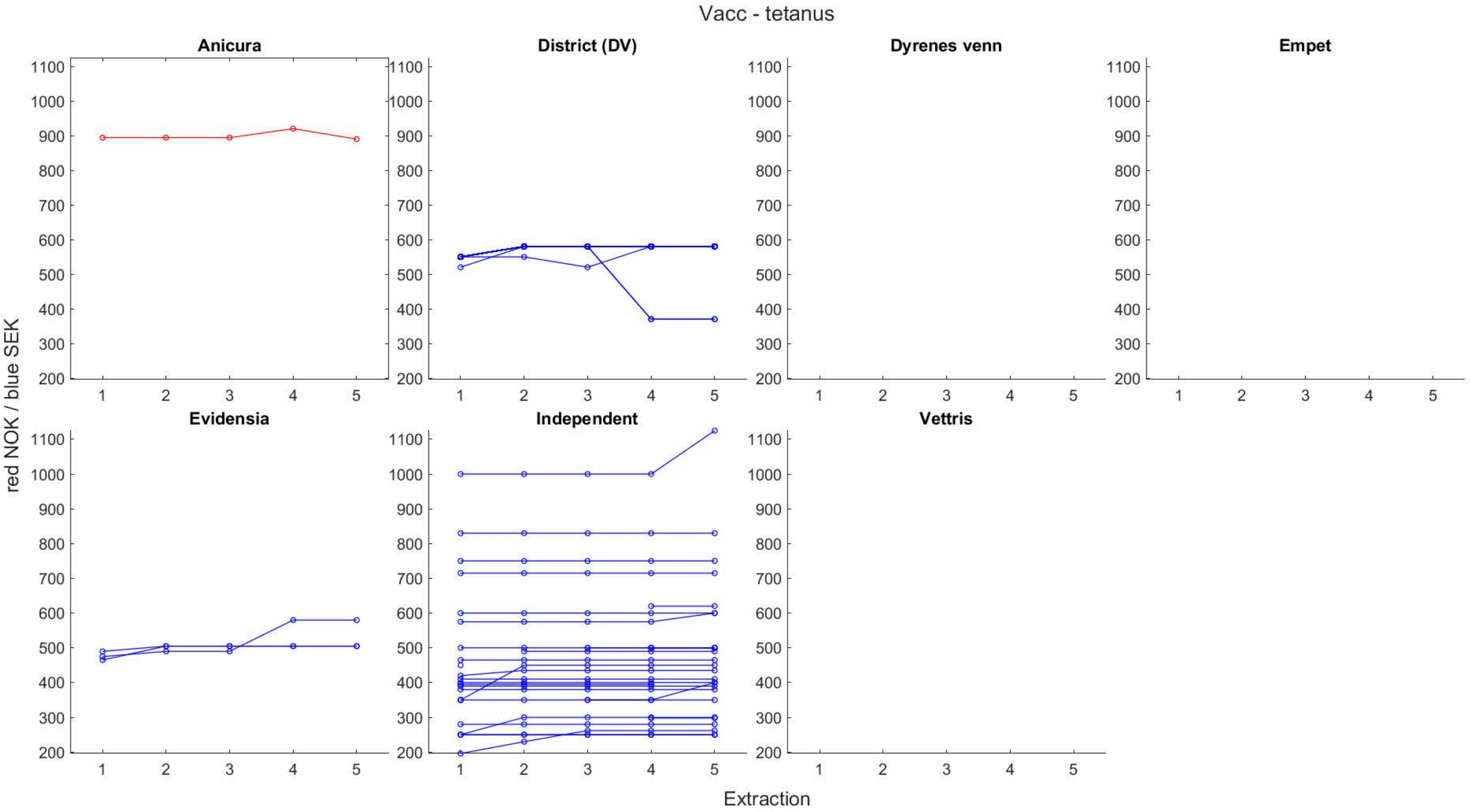

Vacc - tetanus
Anicura
District (DV)
Dyrenes venn
Empet
Evidensia
Independent
Vettris
red NOK / blue SEK
Extraction

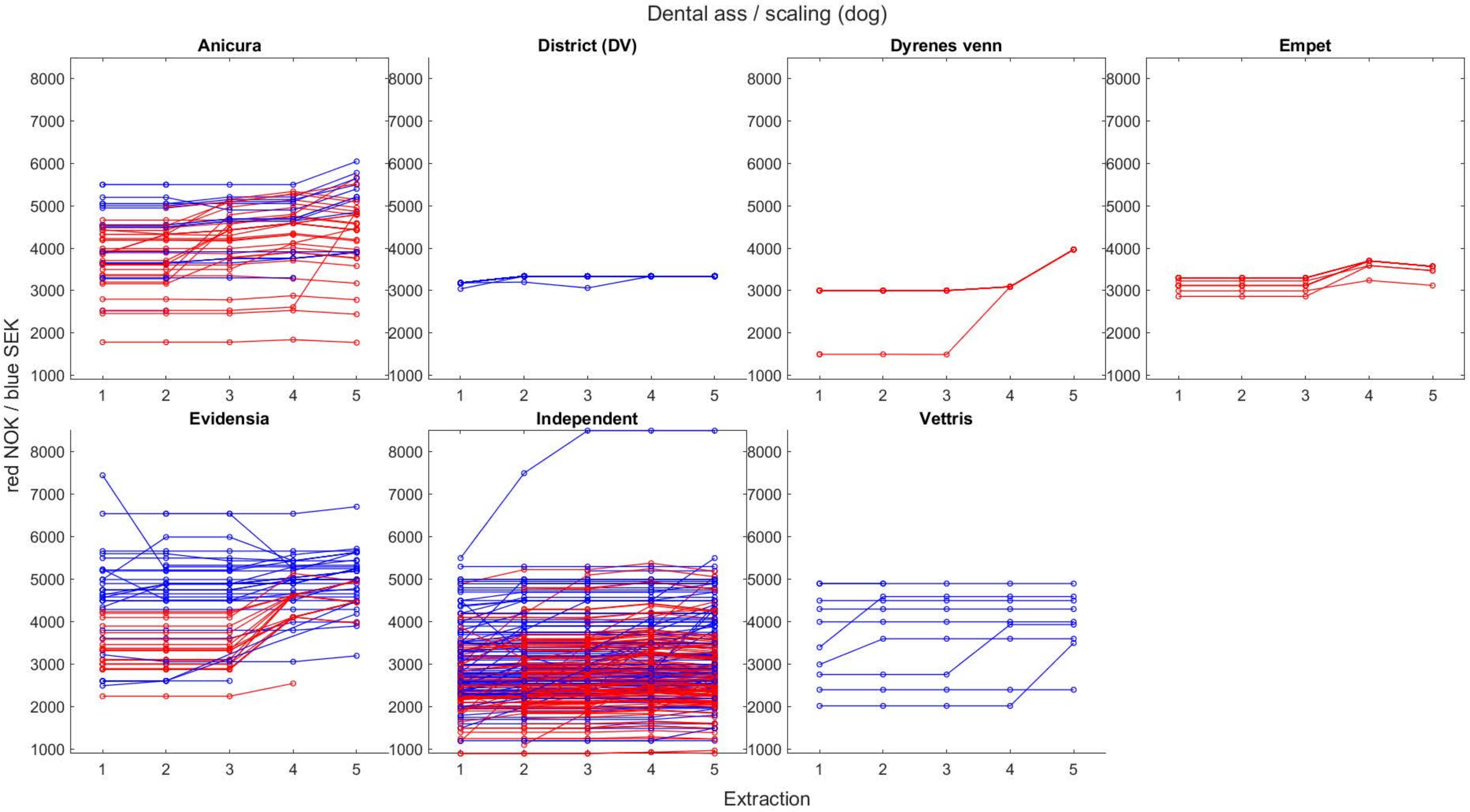

Dental ass / scaling (dog)
Anicura
District (DV)
Dyrenes venn
Empet
Evidensia
Independent
Vettris
red NOK / blue SEK
Extraction
1000
2000
3000
4000
5000
6000
7000
8000
1
2
3
4
5

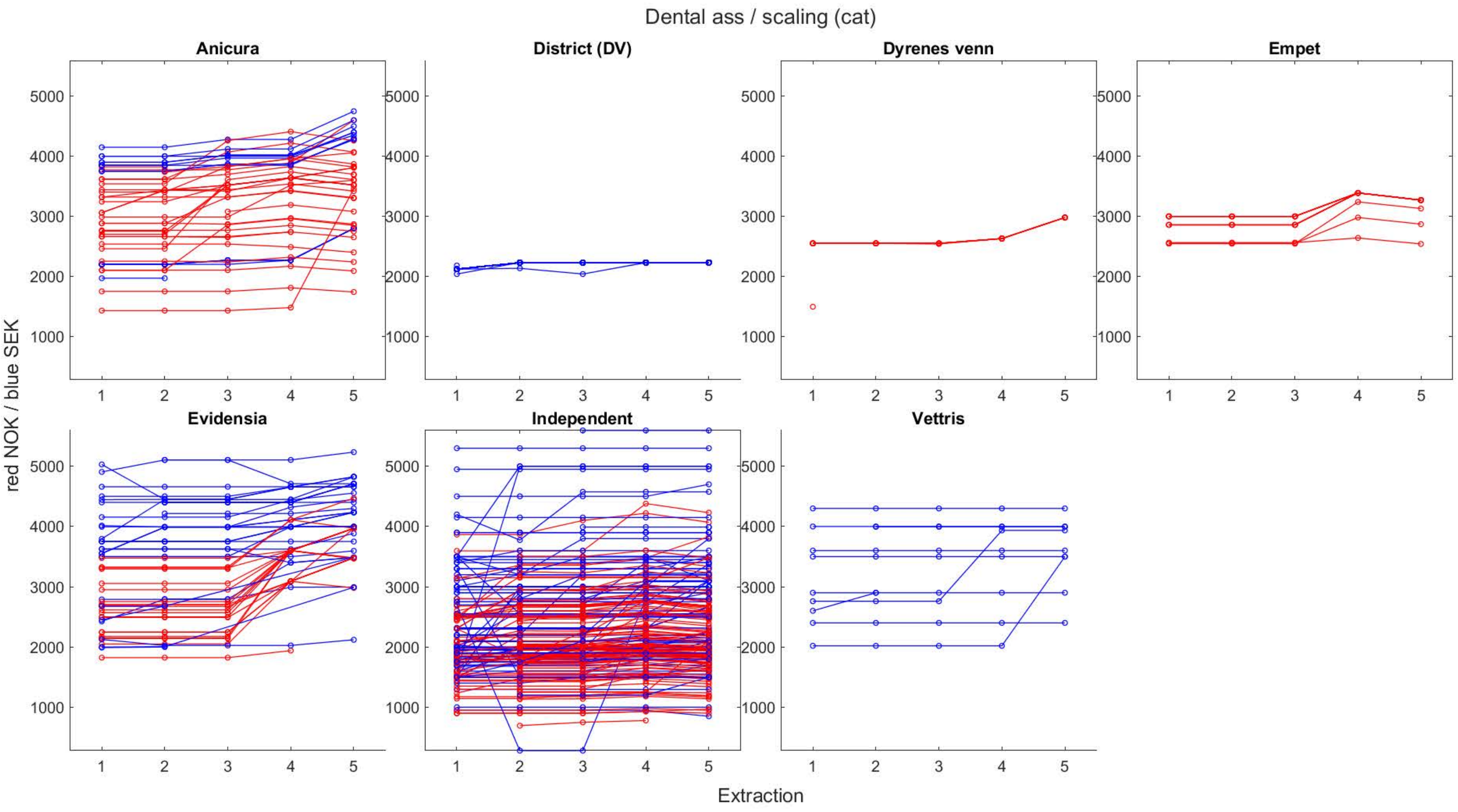

Dental ass / scaling (cat)
Anicura
District (DV)
Dyrenes venn
Empet
Evidensia
Independent
Vettris
red NOK / blue SEK
Extraction

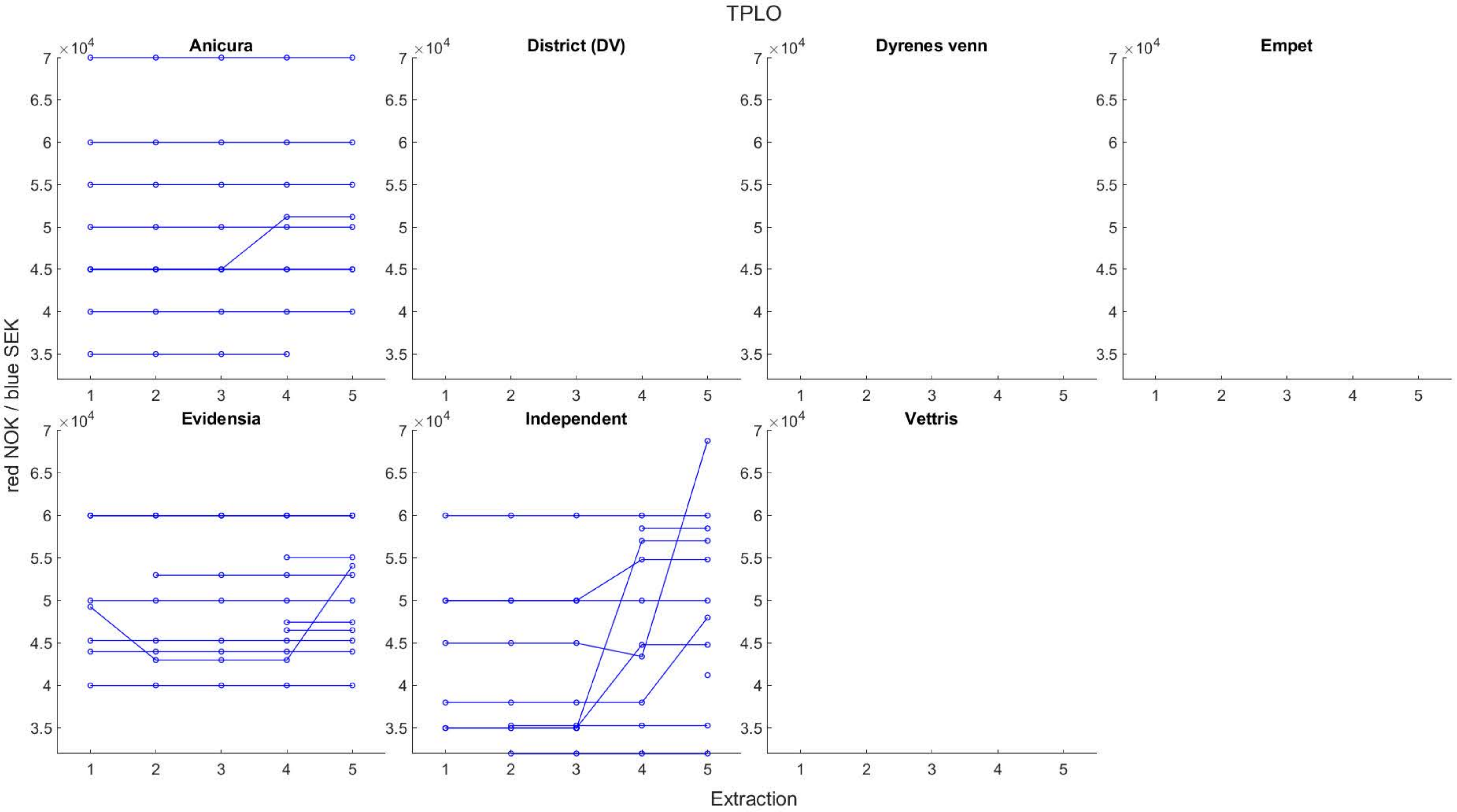

TPLO
Anicura
District (DV)
Dyrenes venn
Empet
Evidensia
Independent
Vettris
red NOK / blue SEK
Extraction

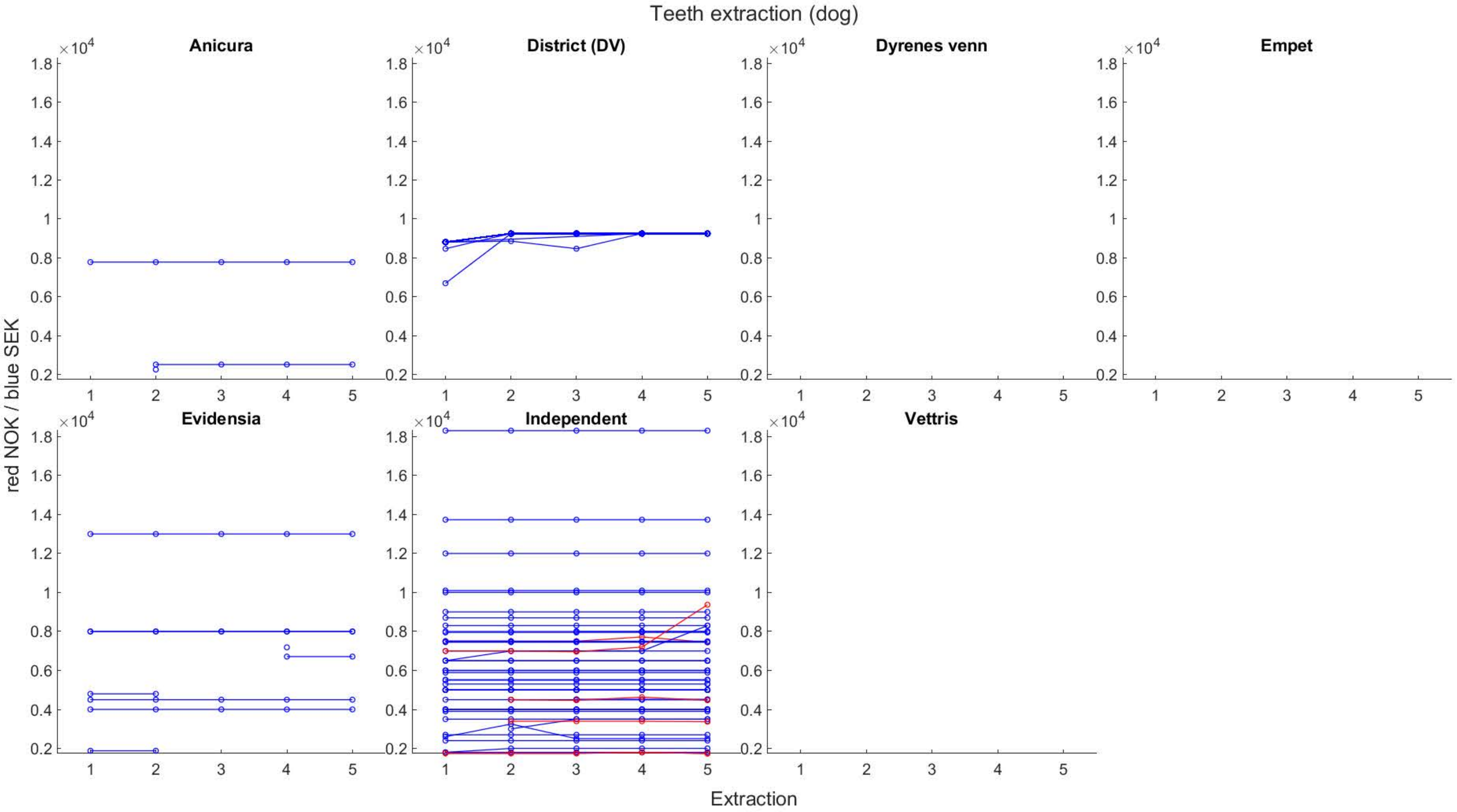

Teeth extraction (dog)
Anicura
District (DV)
Dyrenes venn
Empet
Evidensia
Independent
Vettris
red NOK / blue SEK
Extraction
×10^4
1.8
1.6
1.4
1.2
1
0.8
0.6
0.4
0.2
1
2
3
4
5

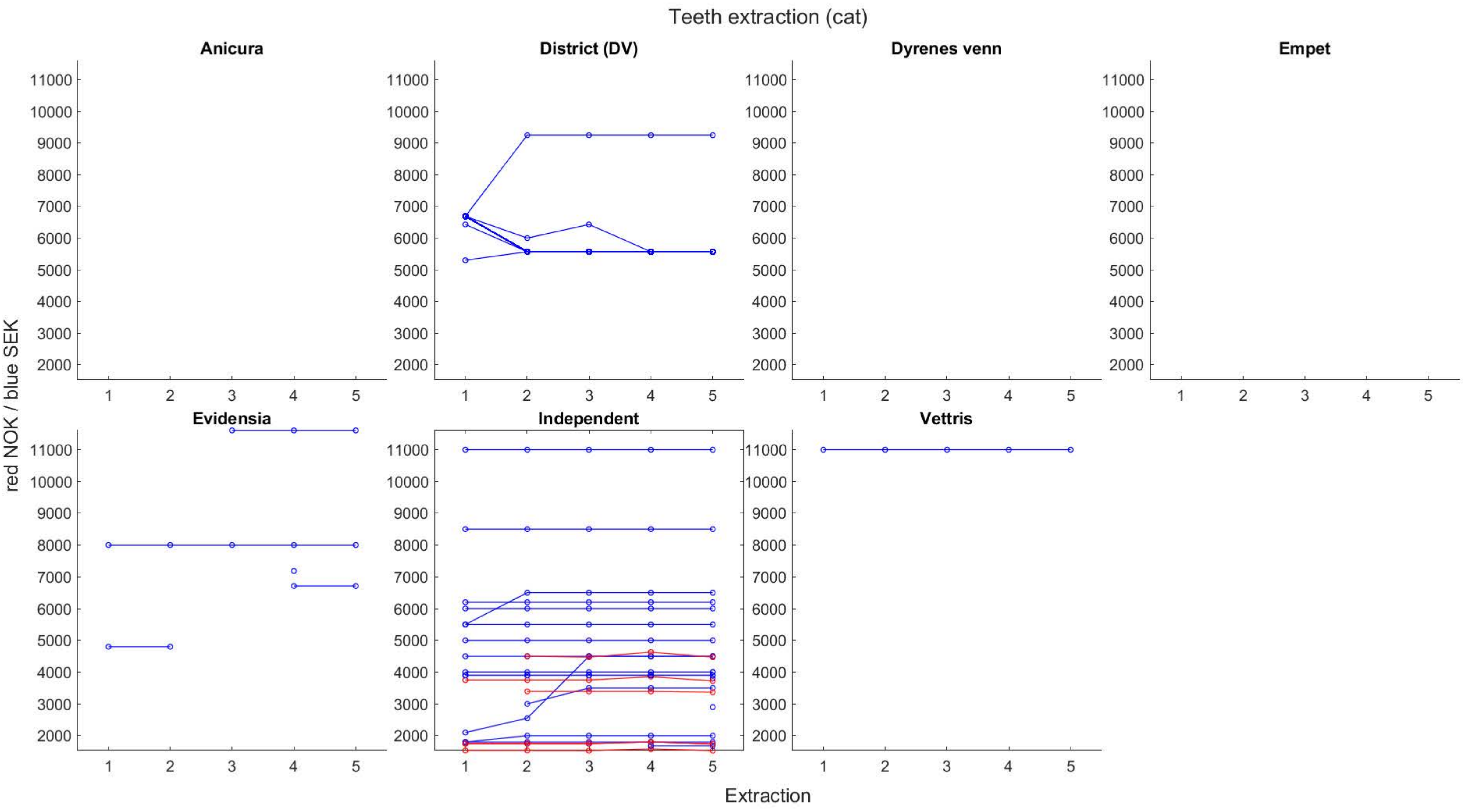

Teeth extraction (cat)
Anicura
District (DV)
Dyrenes venn
Empet
Evidensia
Independent
Vettris
red NOK / blue SEK
Extraction

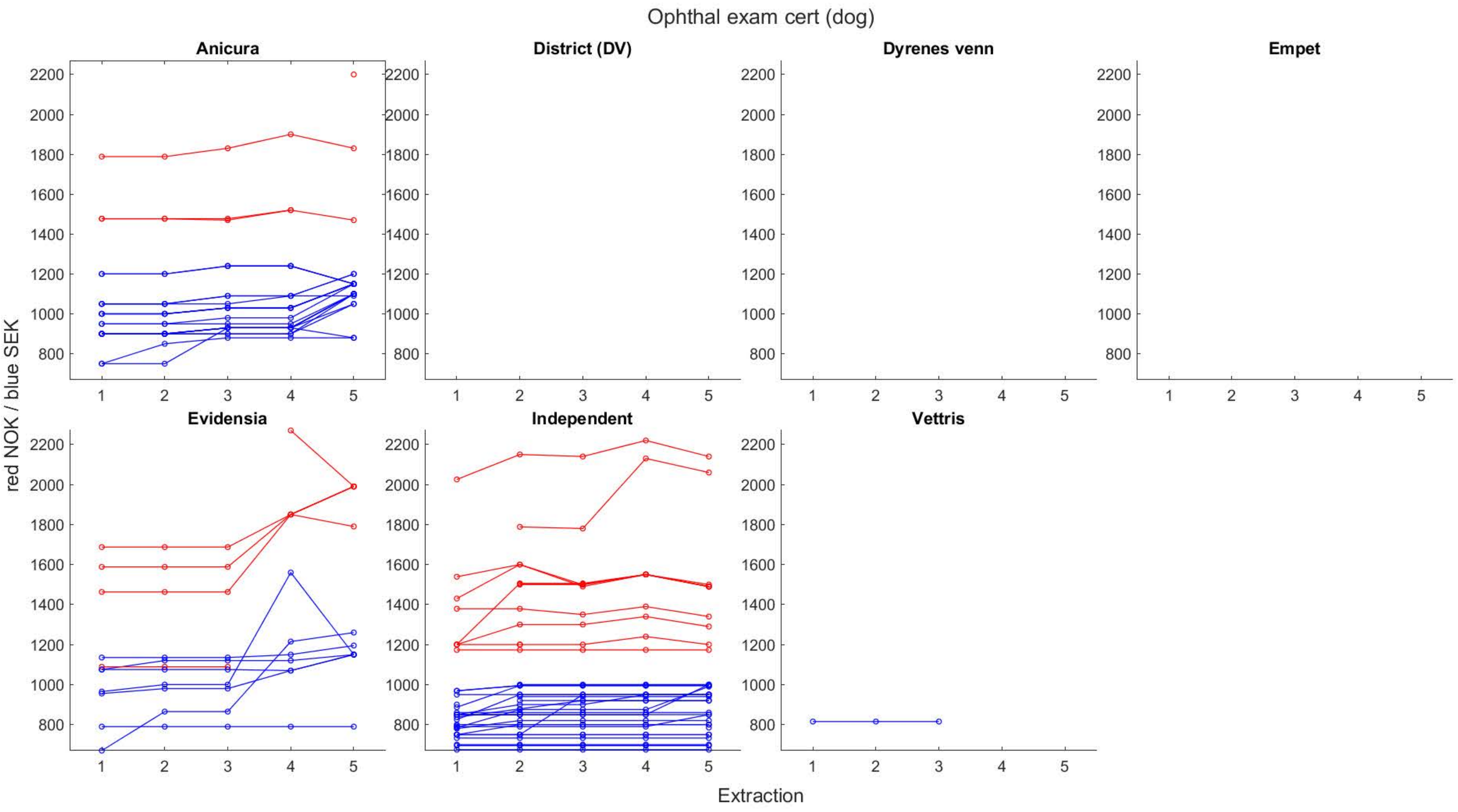
Ophthal exam cert (dog)
Anicura
District (DV)
Dyrenes venn
Empet
Evidensia
Independent
Vettris
red NOK / blue SEK
Extraction
800
1000
1200
1400
1600
1800
2000
2200
1
2
3
4
5

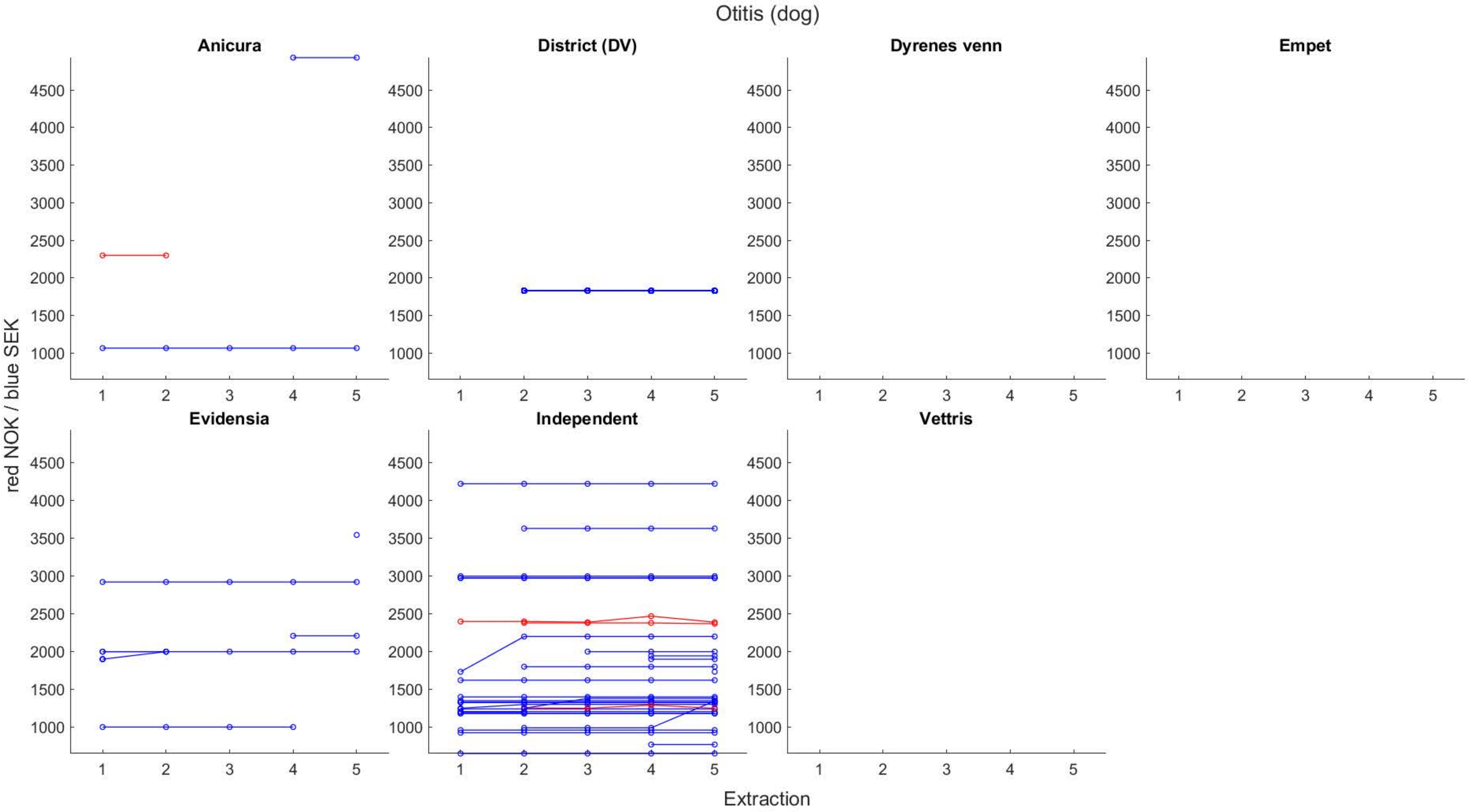

Otitis (dog)
Anicura
District (DV)
Dyrenes venn
Empet
Evidensia
Independent
Vettris
red NOK / blue SEK
Extraction
1000
1500
2000
2500
3000
3500
4000
4500
1
2
3
4
5
